# Direct production of olefins from syngas with ultrahigh carbon efficiency

**DOI:** 10.1038/s41467-022-33715-w

**Published:** 2022-10-10

**Authors:** Hailing Yu, Caiqi Wang, Tiejun Lin, Yunlei An, Yuchen Wang, Qingyu Chang, Fei Yu, Yao Wei, Fanfei Sun, Zheng Jiang, Shenggang Li, Yuhan Sun, Liangshu Zhong

**Affiliations:** 1 CAS Key Laboratory of Low-Carbon Conversion Science and Engineering, Shanghai Advanced Research Institute, Chinese Academy of Sciences, Shanghai, 201210 P. R. China; 2 University of the Chinese Academy of Sciences, Beijing, 100049 P. R. China; 3 School of Physical Science and Technology, ShanghaiTech University, Shanghai, 201210 P. R. China; 4 Shanghai Institute of Applied Physics, Chinese Academy of Sciences, Shanghai, 201800 P. R. China; 5 Shanghai Synchrotron Radiation Facility, Shanghai Advanced Research Institute, Chinese Academy of Sciences, Shanghai, 201210 P. R. China

**Keywords:** Heterogeneous catalysis, Physical chemistry, Materials for energy and catalysis, Chemical engineering

## Abstract

Syngas conversion serves as a competitive strategy to produce olefins chemicals from nonpetroleum resources. However, the goal to achieve desirable olefins selectivity with limited undesired C1 by-products remains a grand challenge. Herein, we present a non-classical Fischer-Tropsch to olefins process featuring high carbon efficiency that realizes 80.1% olefins selectivity with ultralow total selectivity of CH_4_ and CO_2_ (<5%) at CO conversion of 45.8%. This is enabled by sodium-promoted metallic ruthenium (Ru) nanoparticles with negligible water-gas-shift reactivity. Change in the local electronic structure and the decreased reactivity of chemisorbed H species on Ru surfaces tailor the reaction pathway to favor olefins production. No obvious deactivation is observed within 550 hours and the pellet catalyst also exhibits excellent catalytic performance in a pilot-scale reactor, suggesting promising practical applications.

## Abstract (web summary)

Zhong et al report sodium-promoted metallic Ru nanoparticles for the direct production of olefins from syngas with ultrahigh carbon efficiency where the total selectivity of undesired CH_4_ and CO_2_ is suppressed to only 5% for over 500 hours on stream.

## Introduction

Olefins including lower olefins (C_2–4_^=^) and long-chain olefins (C_5+_^=^, olefins with five or more carbon atoms) are important feedstocks in chemical industry for the production of plastics and basic chemicals^1^. Commercially, lower olefins are mainly produced by cracking of naphtha or pyrolysis of light alkanes, while oligomerization of lower olefins leads to high value-added long-chain olefins^2–4^. The limited petroleum resources and the growing market demand for olefins stir the development of alternative routes for olefins production from nonpetroleum feedstocks. Fischer-Tropsch to olefins (FTO) is a highly efficient technology to produce olefins directly from syngas — a mixture of hydrogen and carbon monoxide derived from coal, natural gas, biomass, solid waste, and CO_2_ through commercially mature gasification/reforming technology^5^. However, the goal to achieve desirable olefins selectivity, especially for C_5+_^=^ slate, with limited undesired C1 by-products remains a grand challenge.

**Fig. 1:**
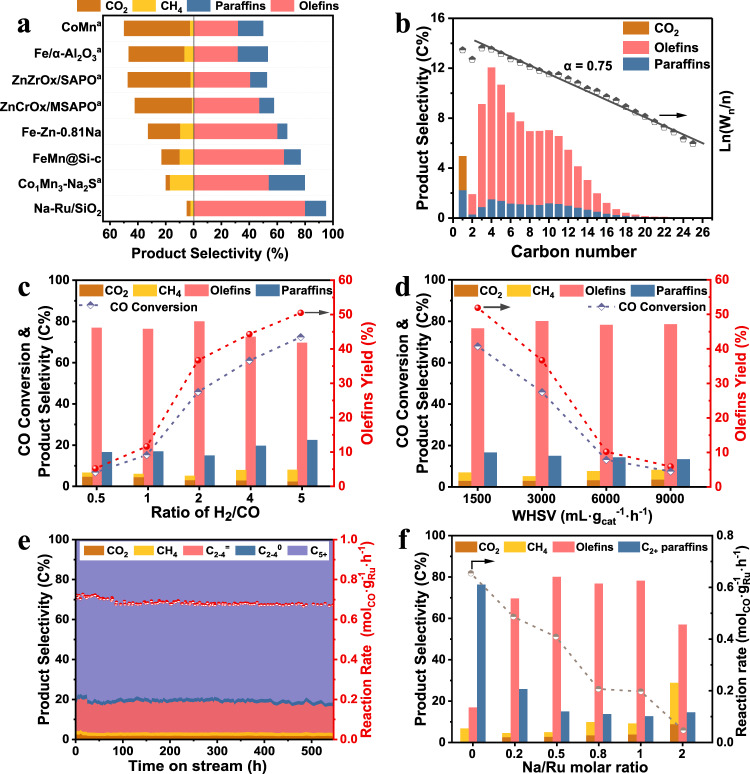
Catalytic performance for direct syngas conversion to olefins. **a** Comparison of catalytic performance among Na-Ru/SiO_2_ and other previously reported catalysts^7–11,13,15^. (a: C_2–4_^=^ selectivity). **b** Detailed product distribution (including CO_2_) and ASF distribution of hydrocarbons over Na-Ru/SiO_2_ catalyst. **c** Product selectivity, CO conversion and olefins yield at different H_2_/CO ratios in syngas over Na-Ru/SiO_2_ catalyst at 533 K, 3000 mL·g_cat._^−1^·h^−1^_,_ and 1.0 MPa. **d** Product selectivity, CO conversion and olefins yield at different space velocities over Na-Ru/SiO_2_ catalyst at 533 K, H_2_/CO ratio of 2 and 1.0 MPa. **e** Stability test for Na-2%Ru(P)/SiO_2_ catalyst. **f** Reaction rate of CO and product selectivity at different Na/Ru molar ratios. Reaction conditions: 533 K, 1.0 MPa, 3000 mL·g_cat._^−1^·h^−1^, H_2_/CO ratio of 2.

Recently, direct conversion of syngas to olefins (STO) has been well explored with significant progress. One of the highlighted routes is based on the bifunctional catalysis using oxide-zeolite composite catalyst (OX-ZEO)^6–8^, where CO activation and C-C coupling are performed on separated active sites, enabling selectivity to lower olefins up to ~80% in hydrocarbons with CO conversion less than 20% under 673 K. Fischer-Tropsch to olefins (FTO) provides another direct route for olefins production from syngas^1,2,9–11^, which is highly efficient for long-chain olefins production. The classic Fischer-Tropsch synthesis (FTS) process mainly produces heavy saturated hydrocarbons over a variety of metal catalysts including iron, cobalt, and ruthenium^12^. To date, only promoted iron- or cobalt carbide catalysts can effectively catalyze FTO reaction with selectivity to lower olefins in hydrocarbons up to 60%^9,13,14^. Nevertheless, both OX-ZEO and metal carbide-based FTO processes commonly exhibit high selectivity to CO_2_ by-products in the range of 30%~50%, significantly decreasing the carbon utilization efficiency^7,11,15,16^. The olefins selectivity reported above would decline to below 60% when being calculated by considering the presence of CO_2_ (Fig. 1a and Supplementary Table 1). In addition, from the viewpoint of practical application, the large amount of CO_2_ produced during STO process not only decreases CO conversion, therefore needing a higher recycle rate to obtain high overall CO conversion, but also requires decarburizing unit to separate the generated CO_2_ in the recycling gas, leading to additional energy consumption in the whole plant. Many recent works try to address this challenge by tailoring the surface properties of catalysts or developing modified FTS catalysts featuring low intrinsic WGS reactivity^11,15–17^. For example, Xu et al. prepared a hydrophobic core-shell FeMn@Si-c catalyst, which can suppress the total selectivity of CO_2_ and CH_4_ to ~23% while remaining ~65% of olefins selectivity at CO conversion of 56.1%^11^. Xie et al. indicated that a Na/S/Mn modified hcp Co presented 54% of selectivity to lower olefins with 17% of CH_4_ selectivity and <3% of CO_2_ fraction at 1% CO conversion^15^. Despite these promising results, carbon efficiency is still low and the goal to reach the maximum olefins (especially for C_5+_^=^) selectivity and yield while simultaneously minimizing the production of undesired C1 by-products including CH_4_ and CO_2_ at a considerable activity level challenge the current FTO technology.

Herein, we present a silica-supported Ru nanoparticles (NPs) catalyst with sodium (Na) as the promoter (denoting as Na-Ru/SiO_2_), which was highly active for FTO reaction but very inactive for WGS reaction. With various characterizations and surface probe reaction experiments, the Ru metal was demonstrated to be the active phase, and the Na promoter can suppress the reactivity of chemisorbed H atoms on Ru surface sites while greatly promote olefins production. The results of this work demonstrate that the modified-metallic Ru can effectively tune the dominated product distribution from traditional paraffins to value-added olefins with sufficient selectivity and yields to justify the promising potential industrial applications.

## Results

### Catalytic performance

The catalytic performance of Na-Ru/SiO_2_ catalyst with 5% of theoretic weight of Ru loading and 0.5 molar ratio of Na/Ru was evaluated at 533 K, 1.0 MPa and H_2_/CO ratio of 2. An unexpected high olefins selectivity up to 80.1% was achieved at a CO conversion of 45.8% with selectivity to CO_2_ and CH_4_ limited within 2.7% and 2.2%, respectively over Na-Ru/SiO_2_ catalyst. (Fig. 1a and Supplementary Table 1). The as-obtained CH_4_ selectivity was far below the value predicted by the Anderson-Schulz-Flory (ASF) rule, and the chain-growth probability (α) for hydrocarbon products was as high as 0.75, indicating its suitability for long-chain olefins production, in good agreement with the observation of 74.5% of C_5+_^=^ slate in the olefins distribution (Fig. 1b). Specially, the fraction of value-added C_5_-C_11_ α-olefins was as high as 57.8%, which can be used for the production of high-quality lubricant, plasticizer and surfactant, while the fraction of detergent-range C_12_-C_18_ α-olefins reached 16.4% (Supplementary Fig. 1). This type of catalyst is also very suitable for converting methane-derived H_2_-rich syngas to long-chain olefins. A higher H_2_/CO ratio benefited CO conversion, which increased to 72.4% at H_2_/CO ratio of 5, for instance, whereas the olefins selectivity maintained above 70% without significant changes in the fraction of C1 by-products (Fig. 1c, Supplementary Table 2). The investigation of the effect of space velocity on catalytic performance suggested that the single-pass yield of olefins can reach up to 51.9% with 6.7% of C1 by-products selectivity at a CO conversion of 67.9% under 1500 mL·g_cat._^−1^·h^−1^·(Fig. 1d, Supplementary Table 3). The increase of reaction pressure, however, could decrease olefins selectivity from 81.0% at 0.5 MPa to 53.5% at 3 MPa (Supplementary Table 4). Furthermore, the stability test was carried out as shown in Supplementary Fig. 2. The catalytic performance for both Ru/SiO_2_ and Na-Ru/SiO_2_ catalysts remained stable within 50 h of test. Especially, the Na-2%Ru(P)/SiO_2_ catalyst with much lower Ru loading amount (1.8 wt.% Ru, ICP) exhibited high stability for 500 h without any significant loss in activity and selectivity. Overall, the activity remained at around 0.700 mol_CO_·g_Ru_^−1^·h^−1^ with intrinsic TOF of 0.210 s^−1^, and the olefins selectivity in total products kept in the range of 75~80% while that of undesired C1 by-products was always suppressed within 5% (Fig. 1e and Supplementary Fig. 3). Compared with the reported results for current STO catalysts under various CO conversion levels (Fig. 1a and Supplementary Table 1), the as-obtained Na-Ru/SiO_2_ catalyst exhibits the highest olefins (especial for C_5+_^=^) selectivity and yield together with the lowest fraction of undesired C1 by-products including CH_4_ and CO_2_.

Noteworthy, the catalytic behavior of Na-Ru/SiO_2_ is quite different from that of a conventional Ru-based FTS catalyst, which mainly produces saturated hydrocarbons instead of olefins^18–20^. As shown in Fig. 1f and Supplementary Fig. 4, the typical Ru/SiO_2_ catalyst without Na promoter exhibits a very high CO conversion (73.3%) with 76.5% of paraffins selectivity and rather low selectivity to olefins (16.9%). After introducing Na promoter, the activity, chain-growth probability (α), and CH_4_ selectivity decreased greatly, while olefins selectivity surprisingly increased to >70% for the sample with a Na/Ru molar ratio of more than 0.2, which reached a maximum value of 80.1% for the sample with Na/Ru molar ratio of 0.5. Furthermore, the product selectivity was compared at similar conversion levels, as shown in Supplementary Fig. 5. Under similar CO conversion of ~70%, the sample of Na-Ru/SiO_2_ still exhibited high olefins selectivity of ~76% with suppressed C1 by-products, while a large amount of paraffins with selectivity of ~76% were produced over Ru/SiO_2_ case. The detailed comparison of each C-containing hydrocarbon product in Fig. 1b and Supplementary Fig. 4 showed that more primary linear olefins than paraffins were produced for Na-Ru/SiO_2_, suggesting that β-H elimination dominated the carbon chain termination pathway according to the Fischer-Tropsch reaction mechanism (Supplementary Fig. 6) and the secondary hydrogenation of olefins was also significantly inhibited. The catalytic performance at varied reaction temperatures further revealed the higher selectivity and yield for olefins over Na-Ru/SiO_2_ catalyst than its Ru/SiO_2_ counterpart (Supplementary Fig. 7, Supplementary Table 5). However, the excessive Na concentration, i.e., Na/Ru atomic ratio of 2, would decline the olefins selectivity significantly and simultaneously cause C1 by-products fraction to surge to ~30%. Obviously, suitable amount of Na doping plays a vital role in tuning the reaction pathway to favor olefins production with inhibited formation rate of C1 by-products and saturated hydrocarbons. Similar promotion effect for olefins production with high selectivity was also observed for other alkali metal promoters (Li, K, Rb, Cs). The increase of atomic number from Li to Cs caused a decreased trend for CO conversion but inversely promoted the formation of long-chain olefins slate from 72.7% for Li-Ru/SiO_2_ to 83.0% for Cs-Ru/SiO_2_ in olefins distribution (Supplementary Table 6). Overall, the alkali-promoted Ru/SiO_2_ catalysts cause the transformation of catalytic performance from typical FTS regime to non-classic FTO with ultrahigh olefins selectivity and limited C1 by-products, which is rarely reported in the previous studies^18,21,22^.

### Structure characterizations

**Fig. 2:**
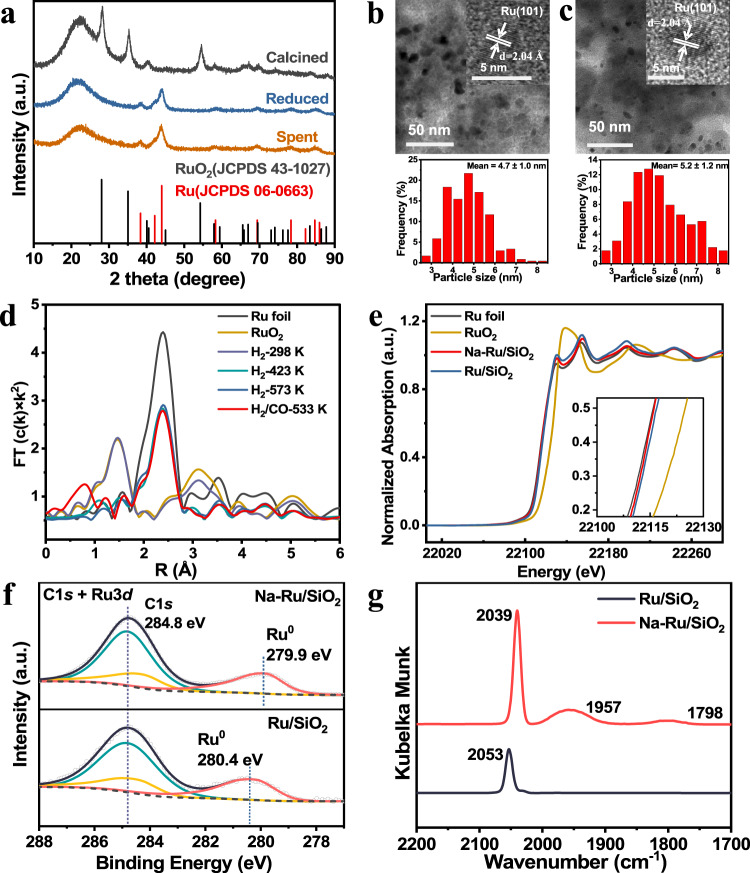
Characterization of Na-Ru/SiO_2_ and Ru/SiO_2_ catalysts. **a** XRD patterns of Na-Ru /SiO_2_ at different stages. **b**, **c** (HR)TEM images and size distribution of Ru NPs for Na-Ru/SiO_2_. **b** Reduced sample; **c** Spent sample. [Insets: Lattice fringes with distance of 2.04 Å corresponding to the Ru (101) crystal plane shown in (**b**, **c**)]. **d** Ru K-edge FT EXAFS spectra under different conditions on Na-Ru/SiO_2_. **e** Ru K-edge X-ray absorption near-edge structure (XANES) spectra of Na-Ru/SiO_2_, Ru/SiO_2_, RuO_2,_ and Ru foil. **f** C 1 *s* and Ru 3*d* photoemission spectra of reduced Ru/SiO_2_ and Na-Ru/SiO_2_. **g** DRIFTS spectra of adsorbed CO on Ru/SiO_2_ and Na-Ru/SiO_2_ at 323 K.

In order to reveal the nature of the active sites that favor the formation of olefins, we resorted to several characterization approaches to investigate the detailed catalyst structure. Ex situ X-ray diffraction (XRD) and in situ XRD results both suggested that RuO_2_ in fresh Na-Ru/SiO_2_ was completely transformed into metallic Ru phase after H_2_ reduction at temperature >573 K, which remained unchanged during the FTO reaction process (Fig. 2a and Supplementary Fig. 8). High resolution transmission electron microscopy (HRTEM) images confirmed the existence of metallic Ru with interplanar distance of (101) at around 2.04 Å and the size of Ru NPs slightly increased from 4.7 ± 1.0 nm to 5.2 ± 1.2 nm after reaction (Fig. 2b, c). Similar metallic Ru phase was observed for spent Ru/SiO_2_ with size of Ru NPs centering at around 8.4 nm (Supplementary Fig. 9). A qualitative comparison of the exposure of Ru species can be obtained by estimating the Ru dispersion through TEM and/or CO chemisorption (Supplementary Table 7). Compared with Ru/SiO_2_, higher metal dispersion (i.e. 11.0%) and metallic surface area (i.e. 49.3 m^2^/g_Ru_) were obtained for Na-Ru/SiO_2_. It was thus suggested that the addition of Na during catalyst preparation could effectively facilitate the dispersion of Ru NPs, in good agreement with XRD &TEM results and previous reports^23,24^. The evolution of the chemical state of Na-Ru/SiO_2_ and Ru/SiO_2_ catalysts during the reduction and FTO reaction process was investigated using in situ X-ray adsorption spectroscopy (XAS). Figure 2d presents the R-space of Fourier transform (FT) extended X-ray adsorption fine structure (EXAFS) spectra at different stages. For the fresh Na-Ru/SiO_2_ sample at room temperature (H_2_-298 K), a major peak corresponding to Ru-O coordination was observed at ~1.5 Å. As the temperature increased to >423 K under H_2_ flow, the peak for Ru-O disappeared. However, the major peak attributed to Ru-Ru pair at ~2.4 Å was observed, closing to that of metallic Ru foil. The above results indicated the complete reduction of RuO_2_ to Ru metal phase. After H_2_-treatment at 573 K, the temperature was decreased to 533 K and the atmosphere was switched to syngas (H_2_/CO = 2) to explore the chemical variation of active Ru centers during the FTO reaction. A very analogous FT EXAFS spectrum was obtained, suggesting an unchanged local geometric structure of the Ru species. The EXAFS fitting results further confirmed the existence of Ru metal phase after reduction and reaction, which is independent of Na doping (Supplementary Fig. 10 and Supplementary Table 8). The change of electronic structure was further analyzed via in situ X-ray absorption near-edge structure (XANES) in Fig. 2e and Supplementary Fig. 11. The absorption edge distinctly shifted toward a lower energy closing to Ru foil for Na-promoted and unpromoted samples, suggesting the reduction of oxidation state of Ru atoms. The similar XANES spectra of Na-Ru/SiO_2_ after reduction and reaction revealed the unchanged metallic state of Ru species, agreeing well with the in situ EXAFS results.

Furthermore, we found that the introduction of Na greatly improved the dispersion of Ru NPs. Specially, the Na promoter was homogenously distributed on both SiO_2_ support and Ru NPs. A higher density of Na promoter can also be clearly identified as Na loading amount increases (Supplementary Fig. 12). The homogeneous distribution of Na over the catalyst surface may benefit the strong electronic interaction between Ru NPs and Na. To confirm this point, we used X-ray photoelectron spectroscopy (XPS) to examine the electronic state of Ru center. As shown in Supplementary Fig. 13, Ru^4+^ was observed for both fresh samples. After reduction, the Ru^0^ 3*d*_5/2_ peak of Na-Ru/SiO_2_ at 279.9 eV exhibited a 0.5 eV lower binding energy than that of Ru/SiO_2_ sample (280.4 eV), implying the increased electronic density over Ru metal phase due to the electron transfer from Na promoter to near-surface Ru^0^ atom (Fig. 2f). The change of Ru electronic state was further demonstrated by CO-DRIFTS spectra (Fig. 2g). The peak at 2053 cm^−1^ was ascribed to linearly coordinated CO on Ru^0^ center over Ru/SiO_2_ sample. However, the peak shifted toward a lower wavenumber at 2039 cm^−1^ for Na-Ru/SiO_2_, reflecting an increase of π* back-donation from Ru atoms to adsorbed CO*^25^. This result confirmed the formation of electron-rich Ru centers for Na-Ru/SiO_2_ sample. Additionally, the peak at 1798 cm^−1^ is ascribed to bridge-bound CO*^26^, and the peak at 1957 cm^−1^ is generally associated with CO adsorbed on Ru-support interface sites^27,28^. The much stronger peak intensity of linearly-bonded CO* and the appearance of another two adsorption peaks for Na-Ru/SiO_2_ catalyst suggested that the presence of Na promoter strengthens the CO adsorption capacity, in line with the observed increased CO uptake for Na-Ru/SiO_2_ than that of Ru/SiO_2_ (Supplementary Table 7). Moreover, the Bader charge analysis based on DFT calculations showed that each Na_2_O moiety donates a total charge of −0.64 |e| to the adjacent Ru atom when loading Na ion on the Ru (0001) surface, making the surface Ru metal species electron-rich (Supplementary Fig. 14).

### Structure-performance relationship

**Fig. 3:**
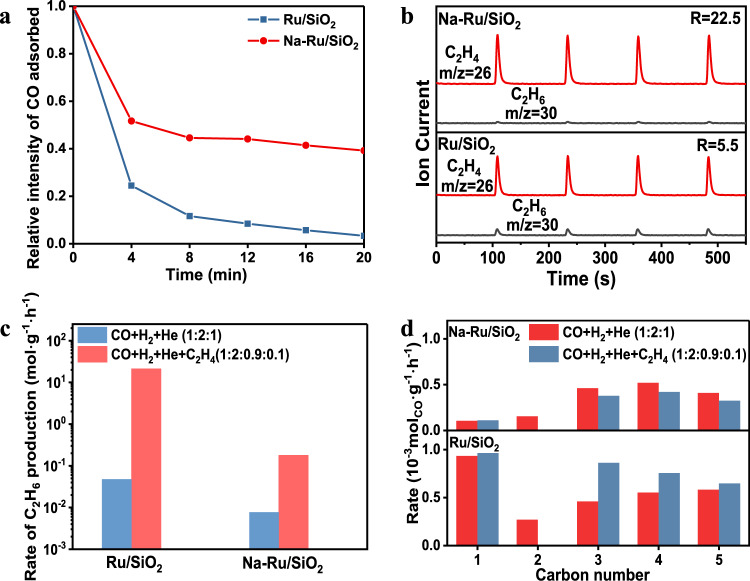
Exploration of the reactivity of chemisorbed H_2_ over Ru/SiO_2_ and Na-Ru/SiO_2_. **a** Relative intensity of linear-adsorbed CO remained during stepwise hydrogenation at 533 K determined by in situ DRIFTS. The ordinates are the evolution of integrated peak area of CO_ad_ species under H_2_ flow divided by that before H_2_ addition at 533 K. **b** Transient response curves obtained during pulses of 370 μL pure C_2_H_4_ into a flow of diluted H_2_ (10% H_2_, 90% Ar, 20 mL·min^−1^) at 533 K. R denotes the integrated peak area ratio of C_2_H_4_/C_2_H_6_ detected by mass spectrometer. **c** Comparison of production rate of C_2_H_6_ before and after the addition of ethene in syngas feedstock, and (**d**) Effect of the ethene co-feeding upon the formation rate of the hydrocarbons products based on a carbon basis at 533 K and 0.5 MPa.

Based on the linear characteristic of ASF distribution and the higher chain-growth probability (α) as well as the ultralow CH_4_ selectivity, it can be inferred that both Ru/SiO_2_ and Na-Ru/SiO_2_ might follow the analogous reaction mechanism^26,29,30^. This can be rationalized from the simplified surface carbide mechanism (Supplementary Fig. 6), which is widely accepted for the FTS^31^. Typically, the dissociated CO would be hydrogenated to form CH_x_ as the main surface intermediate for chain propagation on the metallic Ru surface. The carbon chain grows by coupling of CH_x_ units to the adsorbed alkyl-chain species. The chain growth is terminated by hydrogenation to produce paraffins or β-hydride abstraction to form olefins. We speculate that the possible reason for the huge difference in the catalytic performance of Ru/SiO_2_ and Na-Ru/SiO_2_, including activity and selectivity may lie in the discrepancy of dynamic of chemisorbed hydrogen^23^, which alters the carbon-chain termination pathway. Specifically, the Na promoter significantly weakens the hydrogenation ability of Ru metal surface, thus β-H elimination dominates the carbon-chain termination pathway with the suppression of secondary hydrogenation of olefins. To verify our hypothesis, we designed several experiments to explore the reactivity and mobility of chemisorbed hydrogen. H_2_-TPR results indicated that the presence of Na would suppress the reduction of RuO_2_ to Ru metal (Supplementary Fig. 15), which can be attributed to the lower abundance of adsorbed H_2_ or the reduced surface mobility of chemisorbed H atoms. The evolution of CO_ad_ species during H_2_ flow at 533 K determined using in situ DRIFTS shows that a larger amount of remaining CO_ad_ species with a lower intensity of CH_4_ signal at approximately 3015 cm^−1^ were observed for Na-Ru/SiO_2_ (Fig. 3a and Supplementary Fig. 16). This result revealed that the Na promoter increases the strength of CO adsorption while hindering the reactivity of chemisorbed H_2_, which can hydrogenate the surface carbon species obtained by CO_ad_ dissociation to form CH_4_. Similarly, the observed boosted peak intensity and higher formation temperature of CH_4_ in CO-TPSR result confirm this point (Supplementary Fig. 17). An ethene pulse transient hydrogenation experiment was further performed. The catalysts were firstly reduced in H_2_ for 2 h and reacted in syngas for 1 h, followed by switching to a H_2_ flow at 533 K. The ethene was then pulsed into the systems and the effluent (ethane or ethene) was detected by mass spectrometer. As shown in Fig. 3b, an obvious larger ethane pulse peak was observed for Ru/SiO_2_ with the peak area ratio of ethene to ethane (R) reached 5.5. However, the formation of ethane was almost totally inhibited for Na-Ru/SiO_2_ case with R value surged to 22.5, in accordance with the observed phenomenon for propene pulse transient hydrogenation experiment (Supplementary Fig. 18). It was thus suggested that the Na promoter might increase the “inertness” of adsorbed H and suppress the secondary hydrogenation of olefins. The dynamic of chemisorbed H_2_ can be further distinguished between Ru/SiO_2_ and Na-Ru/SiO_2_ via the experiment of co-feeding of ethene with syngas at 533 K and 0.5 MPa. As shown in Fig. 3c, a considerable part of added ethene was hydrogenated to ethane with formation rate increased by a factor of 592.8 and the ethene/ethane ratio was as low as 0.8 for Ru/SiO_2_. However, the ethene readily desorbed with ethene/ethane ratio reaching 113.9 for Na-Ru/SiO_2_ (Supplementary Fig. 19). Infrared studies of C_2_H_4_ adsorbed also confirmed the promotional effect of Na on the desorption of C_2_H_4_ at 533 K (Supplementary Fig. 20). Furthermore, the adsorption energy was calculated to be −1.09 eV when ethylene was chemisorbed on top of Ru in the π mode, and the C = C bond length was calculated to be 1.45 Å (Supplementary Fig. 21). By comparison, the adsorption energy of ethylene was predicted to be −0.75 eV upon introducing Na_2_O, and the C = C bond length was shortened to 1.43 Å, indicating that the interaction between ethylene and the Ru surface becomes weaker. It was suggested that Ru could acquire additional electrons from Na, and thus favored the desorption of ethylene as well as suppression of the possible secondary hydrogenation of olefins. In addition, according to catalytic performance with ethene co-feeding as shown in Fig. 3d, the absence of Na promoter in Ru/SiO_2_ can boost the extent of ethene participation in the chain initiation and propagation to form C_3+_ products with the increased formation rate of C_3_-C_5_ hydrocarbons by a factor of ~1.5, while an ignorable effect was found for Na-Ru/SiO_2_ due to the weaker ethene adsorption ability on Na-modified Ru metal surface. Obviously, the Na doping would significantly decrease the sticking coefficients and reactivity of H_2_ on Ru metal surface^23^. Previous works^22,23^ has suggested that the alkali promoter would preferentially occupy the low-coordination corner and edge Ru sites required for the dissociation of chemisorbed H_2_ using dynamic ^1^H NMR spectroscopy. Therefore, the structure sensitive, highly mobile and weakly bound β-state of adsorbed H_2_ disappeared for Na-promoted Ru NPs, which can exchange with the mobile α-state and gas phase H_2_ in a fast way. Furthermore, the above characterization results also suggest that CO-rich Ru surface can be obtained for Na-Ru/SiO_2_ catalyst due to the stronger electronic effect of Na promoter. These strongly adsorbed CO* molecules will occupy a large amount of exposed Ru metal sites and thus lower the surface coverage of H_2_^25^. Such promotional effect of electron-rich metal center caused by doping alkaline promoter is also commonly observed for Fe- or Co-based catalysts for FTO reaction^10,11,15,17,32^. Based on these discussions, it is reasonable to speculate that the Na promoter could decrease the fraction of Ru surface sites available for H_2_ adsorption and reduce the mobility of chemisorbed H_2_, thus decreasing the reactivity of H_2_ and hydrogenation capacity of Ru-based FTS catalysts and rending them very efficient for unsaturated olefins production with limited CH_4_ selectivity. In addition, the suppressed reactivity of chemisorbed H_2_ may also lead to the decrease in catalytic activity.

Another intriguing result for the Na-Ru/SiO_2_ catalyst is its strong tendency in hindering the formation of CO_2_ with selectivity normally less than 3.0%, which has long been regarded as a great challenge for traditional STO catalytic systems^11^. In view of the ultralow intrinsic WGS reactivity of metallic Ru, we inferred that the Na-promoted Ru catalyst with metallic Ru as active phase possessed similar property. To verify this viewpoint, a WGS reaction probe experiment was performed and shown in Supplementary Fig. 22. After reaching a steady-state FTO performance, H_2_O stream was introduced into the working reactor. It was found that the TOF value for CO conversion and CO_2_ selectivity remained almost unchanged with the value of ~ 0.102 s^−1^ and 2.1%, respectively. Subsequently, H_2_ flow was turned off, leaving CO and H_2_O as feedstock. Surprisingly, the TOF decreased evidently to 0.005 s^−1^, suggesting that the CO_2_ footprint produced via WGS reaction route can be almost disregarded. Furthermore, the Na doping also slightly increased the CO_2_ selectivity for Ru/SiO_2_ catalyst (Fig. 1f). Prior studies have revealed that the H-assisted CO_ad_ dissociation route prevails on Ru cluster surface with near-saturation CO^*^ coverage during FTS process^26^, which features the preferential formation of H_2_O instead of CO_2_ as the primary oxygen removing pathway (Supplementary Fig. 23), similar to those observed over metallic Co-based catalysts^15^. The suppressed reactivity of chemisorbed H_2_ and increased CO adsorption might slightly benefit the generation of CO_2_ after introducing Na to the Ru/SiO_2_ catalyst.

### Industry-relevant testing

To expand maximumly the industrial application potential of the Na-Ru/SiO_2_ catalyst, the utilization efficiency of noble Ru metal should be further improved. We found that the addition of polyvinylpyrrolidone (PVP) during the catalyst preparation procedure can greatly increase the Ru metal dispersion with higher exposed metallic surface area (Supplementary Table 7), which will significantly boost the production of olefins from syngas for a specific amount of Ru (Supplementary Table 9). The added PVP was completely removed during the calcination process (Supplementary Fig. 24), and the as-obtained catalysts were defined as Na-*y*Ru(P)/SiO_2_, where *y* refers to the theoretical weight percent of Ru. Compared to Na-5%Ru/SiO_2_, the reaction rate of CO over Na-5%Ru(P)/SiO_2_ was significantly elevated to 0.702 mol_CO_·g_Ru_^−1^·h^−1^, which is about 1.5 times higher than the former (Supplementary Table 9). Particularly, the Na-2%Ru(P)/SiO_2_ case with low concentration of Ru loading not only showed perfect catalytic stability (Fig. 1e), but also exhibited a better performance when being evaluated in a pilot-scale fixed-bed reactor (internal diameter: 19 mm; length: 1180 mm) under industrially relevant conditions. As shown in Supplementary Fig. 25, a TOF value of 0.312 s^−1^ was acquired for the pellet catalyst with 72.5% of olefins selectivity together with 1.8% CH_4_ selectivity and 2.5% CO_2_ selectivity, which is similar to that evaluated in a microreactor and superior to most other Ru-based catalysts reported previously (Supplementary Table 10). Compared to previously industrial-favored Fe-based FTO catalyst, the Ru-based case simultaneously exhibits excellent olefins selectivity (~60% vs ~80%), olefins yield (~36% vs ~52%), and catalytic stability under very mild reaction conditions (i.e., 593~613 K vs 533 K, 2~3 MPa vs 1.0 MPa). Moreover, the Na-Ru/SiO_2_ catalyst is more beneficial to the production of value-added long-chain olefins. Future researches are required to further decrease the Ru loading and catalyst cost from the viewpoint of economic feasibility.

## Discussion

In conclusion, we have developed a Na-promoted supported metallic Ru NPs catalyst that can effectively tune the dominated product distribution from traditional paraffins to value-added olefins with high selectivity and yield to justify the promising potential industrial application. The formation of C1 by-products including CH_4_ and CO_2_ is greatly suppressed within <5%. The as-obtained catalyst also significantly benefits the production of long-chain olefins with fraction in produced olefins surpassing 70%, and an excellent catalytic stability is also achieved. Given the technically available approaches for the recovery and recycling of the noble Ru metal, this work establishes brilliant prospect for direct production of olefins, especially for long-chain olefins, from syngas using Ru-based catalysts featuring ultrahigh carbon efficiency.

## Methods

### Catalysts preparation

All catalysts were prepared by the incipient wetness impregnation method. Typically, suitable amount of ruthenium nitrosyl nitrate solution (0.1414 g Ru per gram of solution, Heraeus Precious Metal Technology Co., Ltd.) was diluted with deionized water (13.5 mL) according to the required volume for incipient wetness impregnation of 5.559 g of aerosol silica (SiO_2_, AEROSIL 380, Evonik Degussa China Co., Ltd.). Then different qualities of NaNO_3_ (AR, Sinopharm Chemical Reagent Co., Ltd.) were added into the above ruthenium nitrosyl nitrate solution under vigorous stirring to obtain different Na/Ru molar ratios (0, 0.2, 0.5, 0.8, 1, and 2). Subsequently, the SiO_2_ support was impregnated with the above precursor solution, followed by drying at 333 K for 4 h and calcination in air at 673 K for 4 h. The as-obtained sample was labeled as *x*Na-*y*Ru/SiO_2_, whereas the *x* denotes the molar ratio of Na/Ru, and *y* denotes the theoretic weight of Ru loading. Generally, the sample with *x* value of 0, and *y* value of 5% was abbreviated as Ru/SiO_2_, while the sample with *x* value of 0.5, and *y* value of 5% was abbreviated as Na-Ru/SiO_2_. Specifically, according to above procedure, 2.122 g ruthenium nitrosyl nitrate solution with 0.126 g (1.484 mmol) of NaNO_3_ can obtain the Na-Ru/SiO_2_, while Ru-SiO_2_ was obtained without the addition of NaNO_3_. Both Ru/SiO_2_ and Na-Ru/SiO_2_ samples were mainly used for discussion in this work unless otherwise specified.

The same procedure was carried out for the synthesis of other alkali metal-promoted ruthenium catalysts with 5% of theoretic Ru loading and 0.5 of theoretic molar ratio of alkali promoter to Ru. Typically, 2.122 g ruthenium nitrosyl nitrate solution (0.1414 g Ru per gram of solution, Heraeus Precious Metal Technology Co., Ltd.) was diluted with 13.5 mL deionized water. 1.484 mmol of alkali metal nitrate (LiNO_3_, KNO_3_, RbNO_3_, CsNO_3_) were added into the above ruthenium nitrosyl nitrate solution under vigorous stirring, respectively. Subsequently, 5.559 g of SiO_2_ support was impregnated with the above precursor solution, followed by drying at 333 K for 4 h and calcination in air at 673 K for 4 h. The as-obtained sample was labeled as M-Ru/SiO_2_ (M=Li, K, Rb, Cs).

Moreover, for the PVP-assisted catalyst preparation, the sample was denoted as Na-*y*Ru(P)/SiO_2_ catalyst, whereas the molar ratio of ANa/Ru was also fixed at 0.5, the *y* denotes the theoretic weight of Ru loading, the P denotes the PVP (M_w_ = 58000, Shanghai Aladdin Biochemical Technology Co., Ltd.), and the mass ratio of PVP/Ru was fixed at 10. In a typical synthesis procedure, a certain amount of polyvinylpyrrolidone (PVP) was dissolved in deionized water by vigorous stirring. Then, NaNO_3_ and ruthenium nitrosyl nitrate solution were subsequently added to the dissolved PVP solution. The as-obtained mixed solution was then stirred for 12 h. After that, similar impregnation, drying, and calcination procedure were applied for the preparation of Na-*y*Ru(P)/SiO_2_ as that of *x*Na-*y*Ru/SiO_2_. Specifically, according to above procedure, 2.122 g ruthenium nitrosyl nitrate solution with 0.126 g (1.484 mmol) of NaNO_3_ and 3.295 g PVP can obtain the sample of Na-5%Ru(P)/SiO_2_.

### Catalytic evaluation

Catalysts were evaluated for syngas conversion in a continuous flow fixed-bed reactor with 10 mm inner and an inserted stainless-steel sleeve to monitor the reaction temperature. Typically, 1 g of catalyst sieved into 40 – 60 mesh was diluted with quartz sand (6 g, 40–60 mesh) and loaded into the constant temperature zone of the reactor. Prior to the catalytic reaction, the catalyst was reduced with pure H_2_ (200 mL·min^−1^) at 723 K for 4 h. After the reactor temperature was cooled down, a syngas with a H_2_/CO ratio of 2/1 (H_2_/CO/N_2_ = 64.7/32.3/3) was introduced into the reactor at a flow rate of 50 mL·min^−1^ (Weight hours space velocity (WHSV) = 3000 mL·g_cat._^−1^·h^−1^). The N_2_ was used as internal standard to calculate the CO conversion and product selectivity in the tail gas. The reaction was carried out at 533 K, 3000 mL·g_cat._^−1^·h^−1^, 1.0 MPa, and H_2_/CO ratio of 2 unless otherwise specified. After passing through a hot trap (393 K) and a cold trap (273 K), the gaseous effluent was analyzed online using an Agilent 7890B apparatus equipped with two detectors. A packed column (TDX-01) connected to a thermal conductivity detector (TCD) using He as carrier gas was used to analyze the H_2_, N_2_, CO, CH_4_, and CO_2_. A KCl-modified alumina capillary column (Agilent 19095P-K25) connected to a flame ionization detector (FID) using Ar as carrier gas was used to analyze the hydrocarbons with carbon number in the range of 1–7 (C_1_-C_7_). The aqueous products, liquid oil products, and solid wax products were collected from cold trap and hot trap, and then analyzed off-line with Shimadzu GC. The aqueous products were analyzed via two Porapak Q columns equipped with a TCD for the detection of H_2_O and MeOH and an FID for the detection of C_1_-C_5_ oxygenate. The liquid oil products were analyzed with an HP-1 column connected to an FID using N_2_ as carrier gas. The wax product was dissolved in CS_2_ and analyzed by an MXT-1 column with an FID using N_2_ as carrier gas. The catalytic performance at the stable stage after 12 h of running based on gas analysis was typically used for discussion. The mass balance, carbon balance, and oxygen balance were calculated and maintained at 100 ± 5%. At least three repeated experiments carried out under the same reaction conditions demonstrated that the catalyst shows good reproducibility. Both CO conversion and product selectivity were calculated on a carbon-atom basis. The selectivity of oxygenates was less than 1%, and has been excluded from the reported product selectivity unless otherwise specified.

CO conversion (X_CO_), product selectivity (S_*i*_) and yield (Y_olefins_) were calculated by the following equation:

1 $$\begin{array}{c}{{{{{{\rm{X}}}}}}}_{{{\mbox{CO}}}}=\frac{{F}_{{{{{{\rm{co}}}}}},{{{{{\rm{in}}}}}}}-{F}_{{{{{{\rm{co}}}}}},{{{{{\rm{out}}}}}}}}{{F}_{{{{{{\rm{co}}}}}},{{{{{\rm{in}}}}}}}}\times 100\%\end{array}$$

2 $$\begin{array}{c}{{{{{{\rm{S}}}}}}}_{i}=\frac{{N}_{i}\times {n}_{i}}{\sum ({N}_{i}\times {n}_{i})}\times 100\%\end{array}$$

3 $$\begin{array}{c}{{{{{{\rm{Y}}}}}}}_{{{{{{\rm{olefins}}}}}}}={{{{{{\rm{X}}}}}}}_{{{\mbox{CO}}}}\times {{{{{{\rm{S}}}}}}}_{{{{{{\rm{olefins}}}}}}}\end{array}$$

where F_co,in_ and F_co,out_ represent moles of CO at the inlet and the outlet, respectively, S_*i*_ denotes the selectivity of product *i* on a carbon basis, *N*_*i*_ is the molar fraction of product *i*, and *n*_*i*_ is the carbon number of product *i*, Y_olefins_ denotes the yield of olefins product.

The reaction rate (R) and turnover frequency (TOF) of CO conversion were calculated using the following equations:

4 $$\begin{array}{c}{{{{{{\rm{R}}}}}}}_{{{{{{\rm{CO}}}}}}}=\frac{{V}_{{{{{{\rm{WHSV}}}}}}}\times {{{{{{\rm{X}}}}}}}_{{{\mbox{CO}}}}\times {N}_{{{{{{\rm{CO \, concentration}}}}}}}}{22400\times {\xi }_{{{{{{\rm{Ru}}}}}}}}\end{array}$$

where *V*_WHSV_ is the weight hourly space velocity (mL·g_cat._^−1^·h^−1^), *N*_CO concentration_ denotes the molar concentration of CO in the feedstock, *ξ*_Ru_ denotes the true loading of Ru measured by ICP-AES.

5 $$\begin{array}{c}{{\mbox{TO}}}{{{\mbox{F}}}}_{{{\mbox{CO}}}}=\frac{{{{{{{\rm{R}}}}}}}_{{{{{{\rm{CO}}}}}}}\times {M}_{{{{{{\rm{Ru}}}}}}}}{3600\times {D}_{{{{{{\rm{Ru}}}}}}}}\end{array}$$

where *M*_Ru_ is the atomic mass of Ru (101.07 g·mol^−1^), and the *D*_Ru_ denotes the dispersion of Ru metal measured by CO chemisorption results. The chain growth probability (α) was calculated according to Anderson-Schulz-Flory distribution:

6 $$\begin{array}{c}\left(\frac{{W}_{n}}{n}\right)={(1-{{{{{\rm{\alpha }}}}}})}^{2}{{{{{{\rm{\alpha }}}}}}}^{\left(n-1\right)}\end{array}$$

where *n* is the carbon number of products, *W*_*n*_ is the mass fraction of the hydrocarbons with a carbon number of *n*, and α is chain growth probability.

The equation of (6) can be rewritten as:

7 $$\begin{array}{c}{{{{{\rm{Ln}}}}}}\left(\frac{{W}_{n}}{n}\right)=\left(n-1\right){{{{{\rm{Ln}}}}}}{{{{{\rm{\alpha }}}}}}+2{{{{{\rm{Ln}}}}}}\left(1-{{{{{\rm{\alpha }}}}}}\right)\end{array}$$

Plotting Ln (*W*_*n*_/*n*) versus *n* (carbon number), and the chain growth probability (α) can be obtained by calculating the slope (Lnα).

### Industry-relevant testing

For experiment in industry-relevant testing, 10 g of the pellet catalyst (Na-2%Ru(P)/SiO_2_) with size in cylindrical shape of Φ 5.0 × 3.5 mm was firstly crushed and sieved into size of 12 − 20 mesh, and then diluted with 40 g of SiO_2_, followed by loading into a pilot-scale fixed-bed reactor (internal diameter: 19 mm; length: 1180 mm), which was equipped for operation at industrial working conditions. Before reaction, the catalysts were reduced at 723 K for 4 h in a flow of pure H_2_ with 1000 mL·min^−1^. After that, the temperature was cooled down to 473 K, and the syngas (H_2_/CO/N_2_ = 64.7/32.3/3.0) with a flow rate of 500 mL·min^−1^ was introduced into the reactor and then pressured to 1.0 MPa. The reaction conditions were as follows: 538 K, 1.0 MPa, 3000 mL·g_cat._^−1^·h^−1^, H_2_/CO ratio of 2. The reaction effluent was analyzed with an Agilent 7890B apparatus equipped with two detectors after passing through a hot trap (393 K) and a cold trap (273 K). The liquid products and solid wax products were collected from the cold trap and hot trap, and then analyzed off-line with Shimadzu GC. The identic analysis methods were applied with that investigated in a fixed bed microreactor.

### Catalyst characterization

The Ru concentrations of various reduced samples were measured by inductively coupled plasma optical emission spectrometry (ICP-OES, Perkin Elmer).

Power X-ray diffraction (XRD) data were acquired using a Rigaku Ultima IV X-ray diffractometer (40 kV, 40 mA) equipped with Cu Kα radiation (λ = 1.54056 Å) with scanning angle from 10 to 90° at a scanning speed of 2°·min^−1^. The identification of the structure phase was based on the JCPDS standard card. The XRD crystallite size was calculated by Scherer Formula.

Transmission electron microscopy (TEM) and high-solution transmission electron microscopy (HRTEM) images were obtained on an FEI Tecnai G2 F20 S-TWIN equipment with 200 kV accelerating voltage. Samples for (HR)TEM were prepared by dispersing the sample in ethanol followed by ultrasonication. The nanoparticle size distribution for each sample was determined using ∼300 nanoparticles. High angle annular dark field scanning transmission electron microscopy (HAADF-STEM) images and the corresponding energy dispersion X-ray analysis (EDX) were conducted on a JEOL JEM-F200 microscope equipped with an Oxford EDX detector.

Hydrogen temperature-programmed reduction (H_2_-TPR) was tested on a Micromeritics ChemiSorb2920 with a thermal conductivity detector (TCD). 50 mg of sample was pretreated with He (30 mL·min^−1^) at 393 K for 1 h. After the temperature decreased to 323 K, a 5%H_2_/Ar (30 mL·min^−1^) flow was introduced into the system, and the temperature was ramped from 323 K to 1073 K at a heating rate of 10 K·min^−1^. The signal was recorded by a thermal conductivity detector.

X-ray photoelectron spectroscopy (XPS) was obtained by Thermo Fisher Scientific K-Alpha spectrometer equipped with the Al Kα (1486.6 eV) radiation source. Before XPS measurement, the sample was treated by ion etch to remove surface adsorbate and oxide layer. The results were calibrated by setting the C 1 *s* peak of 284.8 eV.

CO chemisorption experiments were conducted on a Micromeritics ChemSorb2920 with a thermal conductivity detector (TCD). 150 mg catalyst was in situ pretreated with pure H_2_ flow (40 mL·min^−1^) at 723 K for 2 h and then purged with He for 30 min. After the reactor was cooled down to 323 K under He flow, a flow of 10% CO/He was dosed into the reactor until achieving saturated adsorption of CO. Dispersion of Ru was calculated by assuming the stoichiometry of CO/Ru to be 1/1.

In situ diffuse reflectance Fourier transform infrared spectroscopy (DRIFTs) of CO chemisorption experiments were carried out on a ThermoFisher Scientific FTIR spectrometer (Nicolet iS50) equipped with a mercury cadmium telluride (MCT) detector. 20 mg of sample was in situ reduced under a H_2_ flow (40 mL·min^−1^) for 2 h at 723 K. The gas flow was switched to Ar flow to purge the reaction cell for 0.5 h and then the reactor was cooled down to 323 K or 533 K. The background spectra were collected at 323 K or 533 K. Then, Ar flow was switched to CO flow until achieving saturated adsorption. Subsequently, Ar flow was again introduced into the system to remove the gaseous CO, and the DRIFT spectra of CO adsorption were thus collected. As for CO adsorbed (CO_ad_) reactivity experiment at 533 K, after the collection of DRIFT spectra of CO adsorption at 533 K, a flow of diluted H_2_ (10 mL·min^−1^ H_2_ and 30 mL·min^−1^ Ar) was introduced into the cell system, and the DRIFT spectra were automatically recorded to acquire the reactivity of CO_ad_ species. Additionally, the relative intensity of CO adsorbed was compared based on the changes of integrated peak areas at different reaction time at 533 K.

Thermal gravimetric (TG) analysis was performed on a NETZSCH TG/DTA instrument. About 20 mg sample was heated from room temperature to 1073 K in air (40 mL·min^−1^) with 20 mL·min^−1^ Ar (purge gas and shielding gas) at the heating rate of 10 K·min^−1^.

Ethene adsorption experiment was carried out by diffuse reflectance Fourier transform infrared spectroscopy (DRIFTs). 20 mg of sample was reduced in situ under a H_2_ flow (40 mL·min^−1^) for 2 h at 723 K. The gas flow was switched to Ar flow to purge the reaction cell for 0.5 h and then the reactor was cooled down to 533 K. The background spectra were collected at 533 K under Ar atmosphere. Then, the Ar flow was switched to C_2_H_4_ flow until achieving saturated adsorption. Subsequently, the Ar flow was again introduced into the system to remove the gaseous C_2_H_4_, and the DRIFT spectra of C_2_H_4_ adsorption were thus collected.

X-ray absorption fine structure (XAFS) data was performed at the BL14W1 of Shanghai Synchrotron Radiation Facility (SSRF), China. The storage ring of the SSRF was operated at 3.5 GeV with a maximum current of 230 mA. All data were acquired at the Ru K-edge in transmission mode. X-ray absorption near-edge spectroscopy (XANES) and extended X-ray fine-structure (EXAFS) spectroscopy of samples were collected under ambient condition using a fixed-exit double-crystal Si (111) monochromator. The catalyst sample was pressed into pellets within LiF, and then placed inside a stainless steel in situ cell which was surrounded by a heater. Reduction of the Na-Ru/SiO_2_ catalyst was carried out by heating the in-situ cell at 10 K/min in the following pure H_2_ up to 573 K, during which the XAFS spectra were measured at 298 K, 423 K and 573 K, respectively. Then the reduced Na-Ru/SiO_2_ catalyst was treated by syngas (H_2_/CO = 2) in the in-situ cell at 533 K for 30 min and the XAFS spectra was collected. The data analysis was performed using IFEFFIT software package according to standard data analysis procedures^33^. The energy was calibrated by collecting spectra of Ru foil standard sample. After appropriate background subtraction, the *k*^2^-weighted EXAFS spectra of the Ru K-edge data ranges were assessed based on the quality of data generally between k = 3 – 12 Å^−1^ and for R = 1 – 3 Å. All data fitting was performed by Artemis program in IFEFFIT. The value of the passive electron amplitude reduction factor, S_0_^2^, was determined to be 0.75 for Ru, by a fit of a reference Ru foil with a fixed coordination number of 12 to reflect the HCP structure of Ru.

C_2_H_4_ and C_3_H_6_ pulse transient hydrogenation experiments were performed on VDSorb-9Xi instruments equipped with a thermal conductivity detector (TCD) and a MKS Cirrus 2 mass spectrometer. 20 mg of catalyst was loaded into a U-tube reactor and in situ reduced with H_2_ at 723 K for 2 h, and then pretreated under a flow of syngas (H_2_/CO = 2) at 533 K for 1 h, followed by switching to a flow of 10%H_2_/Ar (20 mL·min^−1^). Subsequently, the C_2_H_4_ or C_3_H_6_ was pulsed into the system to complete the pulse transient hydrogenation. The effluent for C_2_H_4_ (m/z = 26) and C_2_H_6_ (m/z = 30), or C_3_H_6_ (m/z = 42) and C_3_H_8_ (m/z = 44) was monitored using a MKS Cirrus 2 mass spectrometer. The integrated peak area ratio of C_2_H_4_/C_2_H_6_ detected by mass spectrometer was calculated and denoted as R, which displays the hydrogenation capacity of different catalysts.

Water-gas-shift (WGS) reaction probe experiment was carried out in a continuous flow fixed-bed reactor with a 10 mm inner diameter. 1 g of Na-Ru/SiO_2_ sample diluted by 6.0 g SiO_2_ was loaded into the reactor and then pretreated by pure H_2_ (50 mL·min^−1^) at 723 K for 4 h. Then, the reactor was cooled down to 533 K, followed by switching a flow of syngas (50 mL·min^−1^, H_2_/CO/N_2_ = 65/32/3, 3 vol.% of N_2_ as internal standard) and then was pressurized to 1.0 MPa. After reaching a steady-state performance according to the analysis of gas phase, a water stream with 0.05 mL·min^−1^ was pumped into the reactor by a high-pressure pump (P230, Dalian Elite Analytical Instruments Co., Ltd, China). The effluent was continuously monitored by an Agilent 7890B chromatograph. After reaching a steady-state performance, the H_2_ flow was turned off, leaving only CO and water as feedstock. The catalytic performance was calculated based on the analysis of the chromatograph data.

Temperature-programmed surface reaction of CO (CO-TPSR) was performed on a Micromeritics ChemiSorb2920 with a thermal conductivity detector (TCD) and MKS Cirrus 2 mass spectrometer. 150 mg of sample was loaded into a U-type reactor and reduced in flow of H_2_ at 723 K for 4 h, followed by flushing with He flow for 30 min. After cooling down to 323 K under He flow, a flow of CO was switched to achieve the saturated adsorption of CO. Subsequently, the gas CO was flushed out from the reactor by He flow. After that, the He stream was replaced by H_2_ flow, and the reactor temperature was heated from 323 K to 773 K at a rate of 10 K·min^−1^. The methane signal (m/z = 14) in the effluent gas was recorded by the mass spectrometer.

The co-feeding of ethene with syngas was employed in a continuous flow fixed-bed microreactor. The effluent gas was analyzed by a gas chromatograph equipped with a packed column (TDX-01) connected to a thermal conductivity detector (TCD) and a capillary column (19095P-K25) connected to a flame ionization detector (FID). 100 mg of Ru/SiO_2_ (80–100 mesh) or 200 mg of Na-Ru/SiO_2_ (80–100 mesh) was diluted with quartz sand (1 g, 80–100 mesh) and then loaded into the quartz tube. The sample was reduced at 723 K for 4 h in a flow of H_2_ at atmospheric pressure. Then, the reactor was cooled down to the reaction temperature (533 K). Syngas (H_2_/CO = 2) with flow rate of 30 mL·min^−1^ and He with flow rate of 10 mL·min^−1^ were introduced into the reaction system and the reactor pressure was also pressurized to 0.5 MPa. After reaching steady-state FTO performance, the He flow was switched to 10%C_2_H_4_/He (10 mL·min^−1^). CO conversion and products selectivity were calculated by an Agilent 7890B apparatus. The reaction was carried out at low CO conversions (<2%) to ensure differential operation.

### Computational details

Periodic density functional theory (DFT) calculations were performed with the Vienna ab initio simulation package (VASP)^34,35^. The Perdew-Burke-Ernzerhof (PBE) exchange-correlation functional^36^ was applied with a plane-wave basis set. A cutoff energy of 400 eV and the projector augmented wave (PAW) method was applied throughout the calculations^37^. A Gaussian smearing method with a width of 0.2 eV was used during iterative diagonalization of the Kohn-Sham Hamiltonian. The electronic energy of the structure was converged to 10^−4^ eV in the self-consistent field calculations, whereas the force on each relaxed atom was converged to 0.03 eV·Å^−1^ in the ionic relaxation calculations. Both the dimer^38^ and the climbing-image nudged elastic band (CI-NEB) method^39,40^ were used to locate the transition states (TS), which were then verified to have one and only one imaginary frequency.

The lattice parameters of hexagonal Ru was calculated to be a = b = 10.82, c = 18.42, which were in good agreement with experimental^41^ and other theoretical values^26^. The Ru (0001) surface with a p (4 × 4) supercell was employed in this work, which consists of 64 atoms in 4 layers. The bottom two layers were fixed to mimic the bulk, while the remaining layers, along with the adsorbates, were allowed to relax. A Γ-centered 3 × 3 × 1 Monkhorst–Pack k-point mesh and a 12Å vacuum layer perpendicular to the slab were used.

## Supplementary information

Supplementary Information

Peer Review File

## Data Availability

The data that support the findings of this study are available within the paper and its Supplementary Information, and all data are also available from the corresponding authors upon reasonable request. Source data are provided with this paper.

## Source data

Source Data
