# Supplementary material for: Direct production of olefins from syngas with ultrahigh carbon efficiency
Source: Nat Commun. 2022 Oct 10;13:5987. doi: 10.1038/s41467-022-33715-w (PMC9550792; doi:10.1038/s41467-022-33715-w)
Supplement: Supplementary file 1 — Supplementary Information [file 41467_2022_33715_MOESM1_ESM.pdf]

Supplementary Information

Direct production of olefins from syngas with ultrahigh carbon efficiency

Hailing Yu[1,2#], Caiqi Wang[1,2#], Tiejun Lin[1#], Yunlei An[1], Yuchen Wang[1,3], Qingyu Chang[1], Fei Yu[1], Yao Wei[2,4], Fanfei Sun[5], Zheng Jiang[5], Shenggang Li[1,3], Yuhan Sun[1,3]*, Liangshu Zhong[1,3]*

[1] CAS Key Laboratory of Low-Carbon Conversion Science and Engineering, Shanghai Advanced Research Institute, Chinese Academy of Sciences, Shanghai 201210, P.R. China

[2] University of the Chinese Academy of Sciences, Beijing 100049, P.R. China

[3] School of Physical Science and Technology, ShanghaiTech University, Shanghai 201210, P.R. China

[4] Shanghai Institute of Applied Physics, Chinese Academy of Sciences, Shanghai 201800, P.R. China

[5] Shanghai Synchrotron Radiation Facility, Shanghai Advanced Research Institute, Chinese Academy of Sciences, Shanghai 201210, P.R. China

*Corresponding author. Email: sunyh@sari.ac.cn; zhongls@sari.ac.cn

[#]These authors contributed equally to this work.

Supplementary Fig. 1 3
Supplementary Fig. 2 4
Supplementary Fig. 3 5
Supplementary Fig. 4 6
Supplementary Fig. 5 7
Supplementary Fig. 6 8
Supplementary Fig. 7 9
Supplementary Fig. 8 10
Supplementary Fig. 9 11
Supplementary Fig. 10 12
Supplementary Fig. 11 13
Supplementary Fig. 12 14
Supplementary Fig. 13 15
Supplementary Fig. 14 16
Supplementary Fig. 15 17
Supplementary Fig. 16 18
Supplementary Fig. 17 19
Supplementary Fig. 18 20
Supplementary Fig. 19 21
Supplementary Fig. 20 22
Supplementary Fig. 21 23
Supplementary Fig. 22 24
Supplementary Fig. 23 25
Supplementary Fig. 24 26
Supplementary Fig. 25 27
Supplementary Table 1 29
Supplementary Table 2 30
Supplementary Table 3 31
Supplementary Table 4 32
Supplementary Table 5 33
Supplementary Table 6 34
Supplementary Table 7 35
Supplementary Table 8 36
Supplementary Table 9. 37
Supplementary Table 10 38
Supplementary References 39

Supplementary Figures

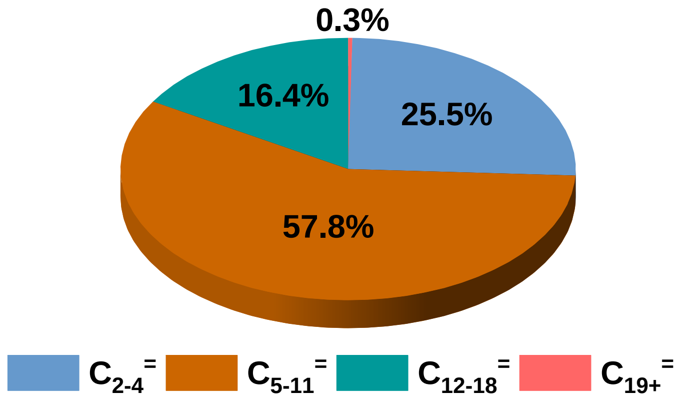

Supplementary Fig. 1 | Detailed olefins distribution of Na-Ru/SiO_2. Olefins distribution in the carbon number range of C_{2-4}, C_{5-11}, C_{12-18} and C_{19+}.

The Na-Ru/SiO_2 catalyst exhibits a narrower carbon distribution compared with the classical FT catalysts. The fraction of lower olefins ($C_{2-4}^{=}$) accounts for 25.5%, which is commonly used for bulk chemicals. While the fraction of C_{5-11} olefins reaches 57.8%, and can be widely used as raw materials and/or intermediates for production of chemicals such as lubricant, plasticizer and surfactant. In addition, the C_{12-18} slate olefins with fraction of 16.4% favors the production of detergent.

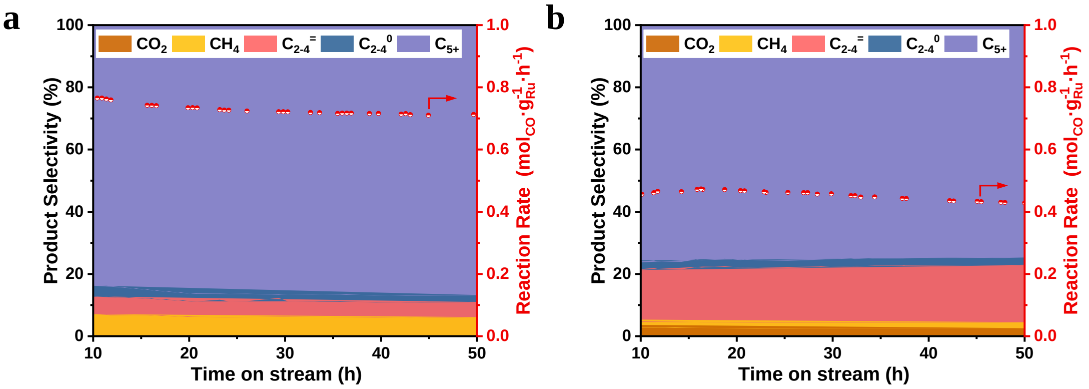

Supplementary Fig. 2 | Stability test. Catalytic performance of Ru/SiO_2 (**a**) and Na-Ru/SiO_2 (**b**) catalysts with time on stream. Reaction conditions: 533 K, 3000 mL $g_{cat.}^{-1}$ h^{-1}, 1 MPa, H_2/CO ratio of 2.

The stability test of Ru/SiO_2 and Na-Ru/SiO_2 was carried out at the same reaction conditions. As can be seen, the catalytic performance for both Ru/SiO_2 and Na-Ru/SiO_2 catalysts remained stable within 50 h.

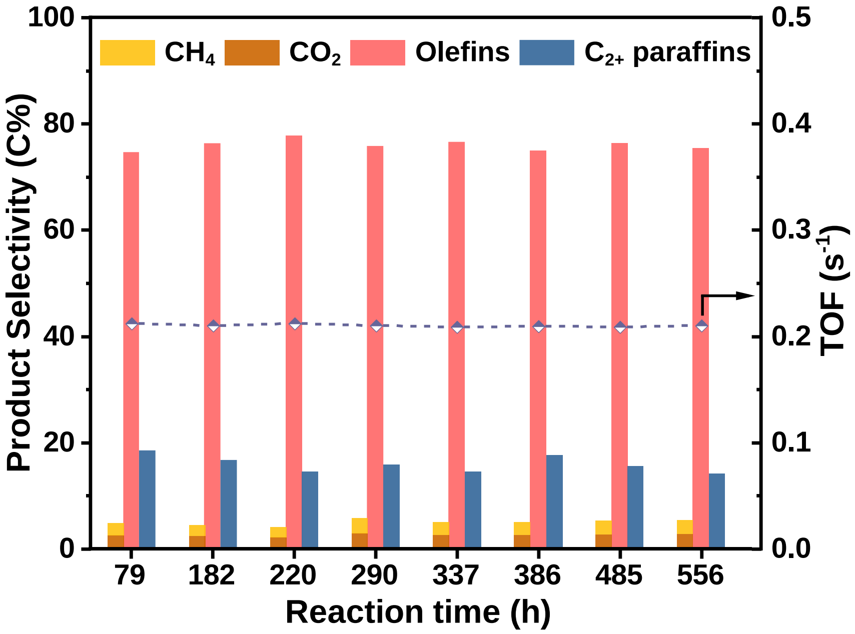

Supplementary Fig. 3 | Stability test of Na-2%Ru(P)/SiO_2 catalyst. Reaction conditions: 533 K, 3000 mL $g_{cat.}^{-1}$ h^{-1}, 1 MPa, H_2/CO ratio of 2.

The reaction effluent of Na-2%Ru(P)/SiO_2 catalyst was analyzed at different time-on-stream and the intrinsic TOF was calculated to be as high as ~0.200 s^{-1}. Excellent stability over Na-2%Ru(P)/SiO_2 was obtained and olefins selectivity remained high in the range of 75-80% along with the ultralow total selectivity to CH_4 and CO_2 (< 5%).

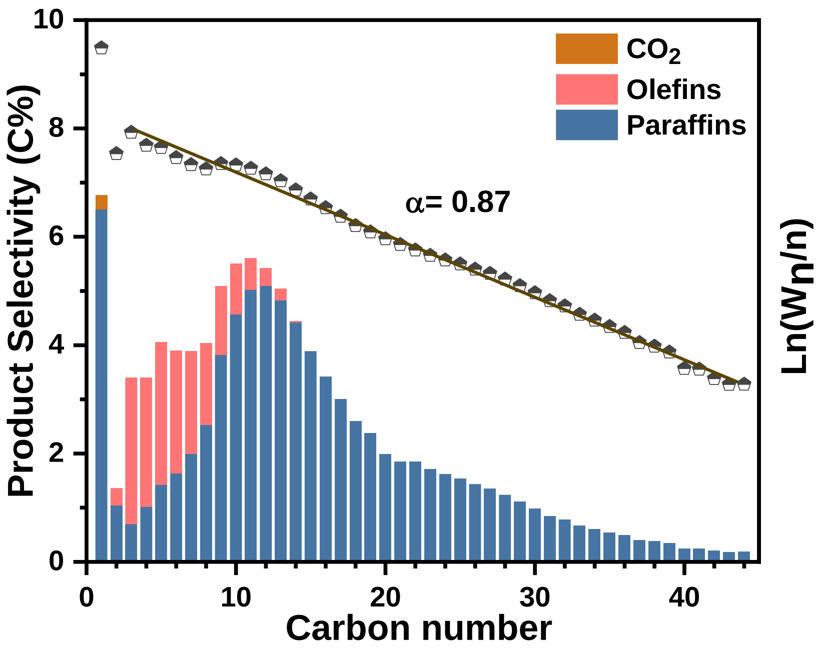

Supplementary Fig. 4 | Product distribution of Ru/SiO_2 catalyst. Detailed product selectivity and the ASF distribution of hydrocarbons over Ru/SiO_2 catalyst. Reaction conditions: 533 K, 3000 mL $g_{cat.}^{-1}$ h^{-1}, 1 MPa, H_2/CO ratio of 2.

As can be seen from Supplementary Fig. 4, the paraffins were the dominated products over Ru/SiO_2 catalyst. The as-obtained linearly carbon distribution followed the Anderson-Schulz-Flory (ASF) rule, and the chain-growth probability (α) for hydrocarbon products was as high as 0.87. This phenomenon suggests that the Ru/SiO_2 catalyst is very suitable for the production of saturated hydrocarbons.

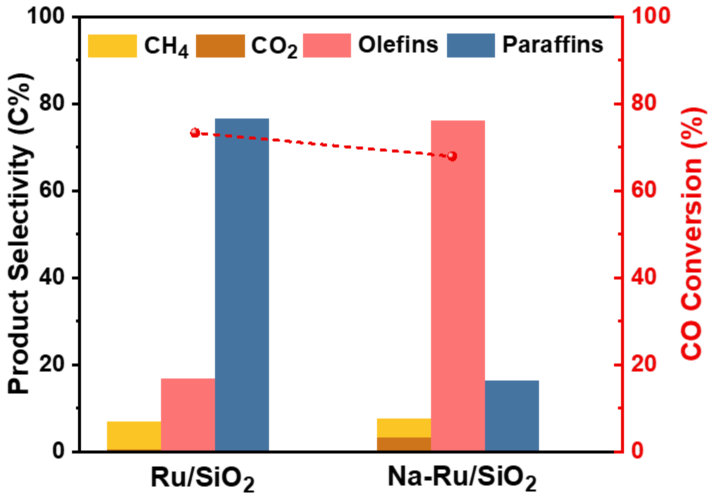

Supplementary Fig. 5 | Comparison of catalytic performance at similar CO conversion level over Ru/SiO$_2$ and Na-Ru/SiO$_2$ catalysts. Reaction conditions: 533 K, 3000 mL $g_{cat.}^{-1}$ h^{-1} (Ru/SiO$_2$), and 1500 mL $g_{cat.}^{-1}$ h^{-1} (0.5Na-Ru/SiO$_2$), 1 MPa, H_2/CO ratio of 2.

The product selectivity was compared at similar high CO conversion (~70%) level for Ru/SiO$_2$ and Na-Ru/SiO$_2$ catalysts. Obviously, the high olefins selectivity (~76%) with limited C1 byproducts selectivity were still maintained even at high CO conversion for Na-Ru/SiO$_2$, while the Ru/SiO$_2$ mainly produced saturated paraffins, demonstrating that the Na-Ru/SiO$_2$ catalyst is suitable for the production of olefins whatever the CO conversion level.

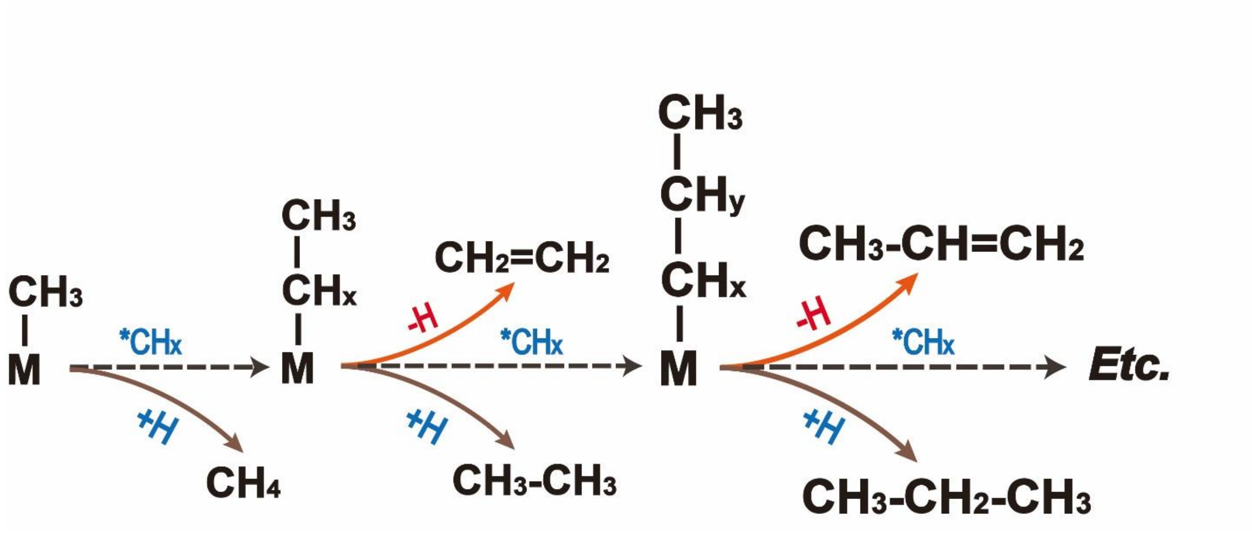

Supplementary Fig. 6 | Chain growth mechanism. The carbon chain growth of Fischer-Tropsch synthesis based on the widely accepted carbide mechanism[1-3], and the chain growth is terminated by β-hydride elimination or hydrogenation.

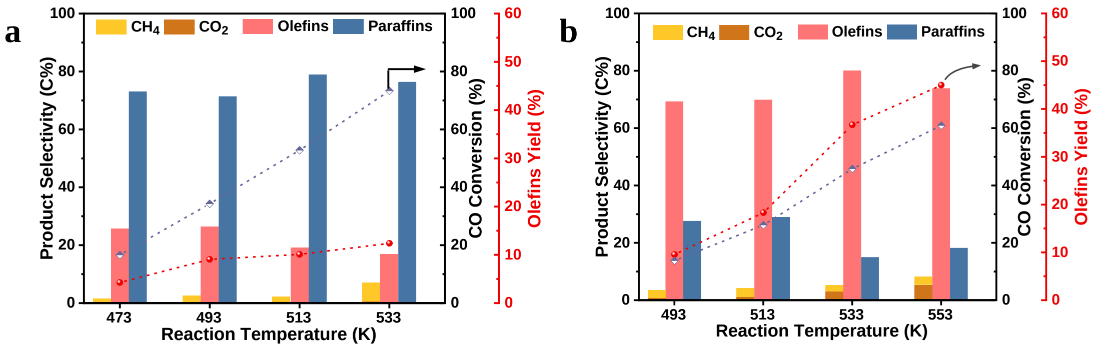

Supplementary Fig. 7 | Effect of reaction temperature. Catalytic performance of Ru/SiO_2 (**a**) and $Na\text{-}Ru/SiO_2$ (**b**) at various reaction temperatures.

As shown in Supplementary Fig. 7, the Ru/SiO_2 and $Na\text{-}Ru/SiO_2$ catalysts showed a quite different product selectivity at any reaction temperature, whereas saturated hydrocarbons dominated the products for the former, while olefins were always the primary products over $Na\text{-}Ru/SiO_2$. Increasing reaction temperature can increase CO conversion for both samples. Compared with Ru/SiO_2, olefins yield was substantially enhanced for $Na\text{-}Ru/SiO_2$ at high reaction temperature. These results suggested that the Na promoter can greatly accelerate olefins production for Ru-based Fischer-Tropsch synthesis.

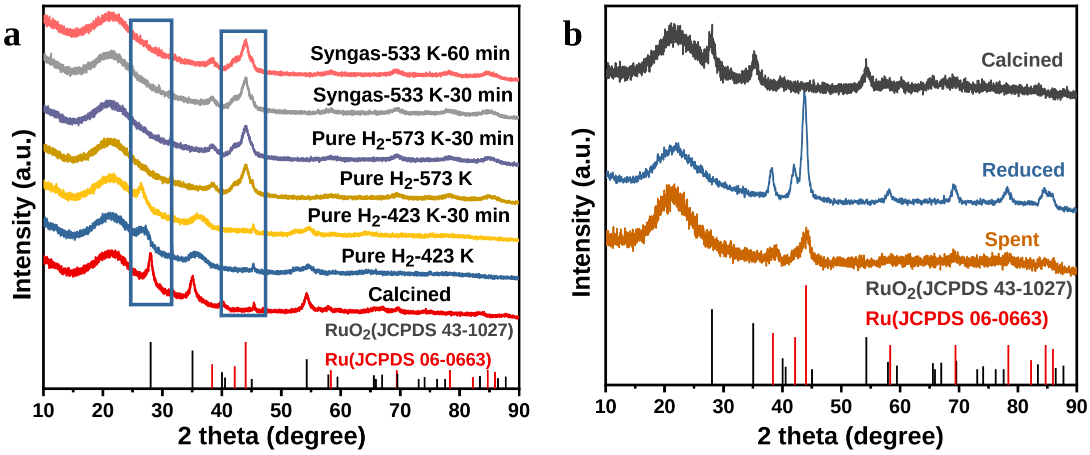

Supplementary Fig. 8 | XRD characterization. (**a**) In situ XRD patterns of Na-Ru/SiO_2 catalyst during stepwise reduction and reaction process at temperature ranging from room temperature to 573 K. (**b**) XRD patterns of Ru/SiO_2 at different stages.

For Na-Ru/SiO_2 catalyst, the phase was gradually transformed from RuO_2 (JCPDS 43-1027) to metallic Ru (JCPDS 06-0663) with the increase of reduction temperature or reduction time. As shown in Supplementary Fig. 8b, the XRD pattern of the reduced sample indicated ruthenium oxide (RuO_2) was completely reduced to metallic Ru (Ru^0). After prolonged exposure to reaction conditions, obvious peaks for Ru^0 phase could be detected for both the unpromoted Ru/SiO_2 and Na-Ru/SiO_2 samples.

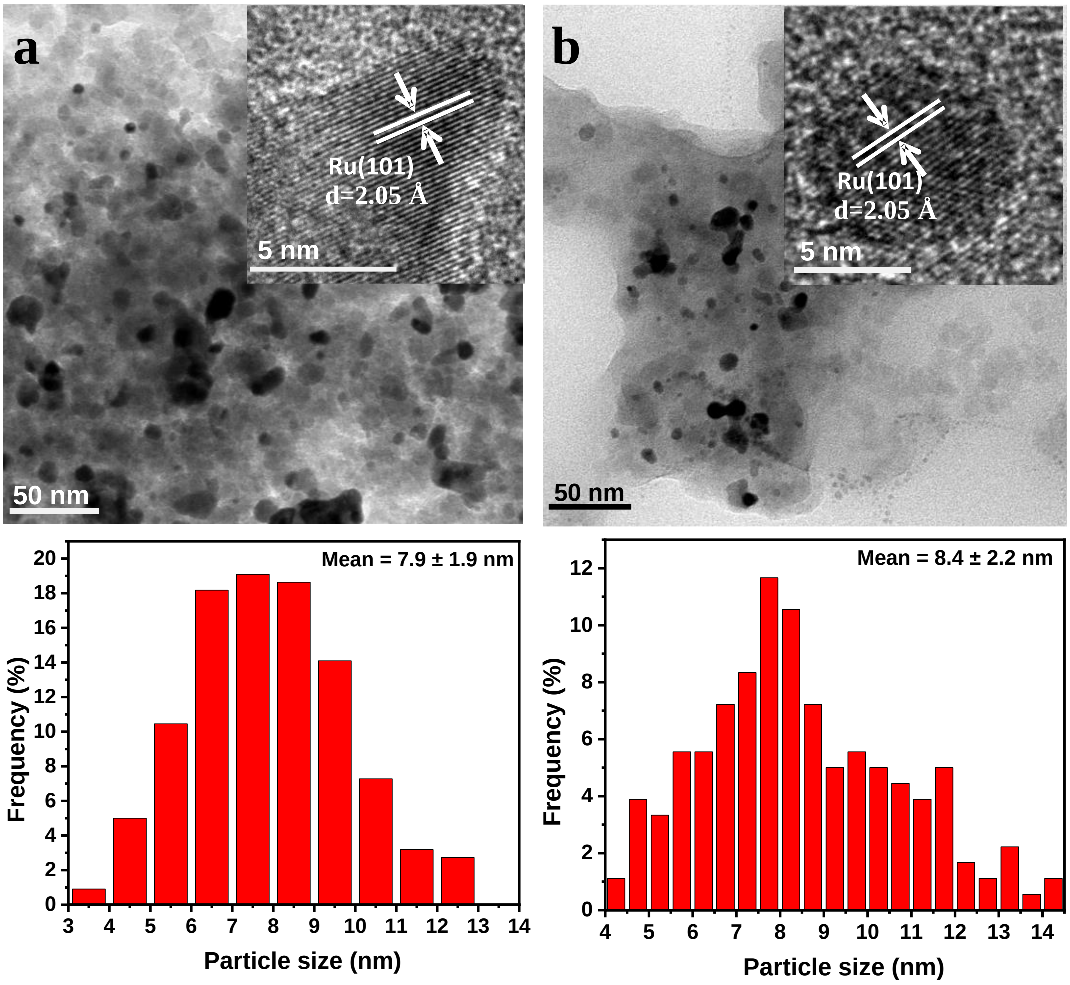

Supplementary Fig. 9 | (HR)TEM characterization. (HR)TEM images and size distribution of Ru nanoparticles for Ru/SiO_2 at different stages. **(a)** Ru/SiO_2 catalyst after reduction at 723 K for 4 h in pure H_2 flow. (**b**) Ru/SiO_2 catalyst after reaction in syngas at 533 K. [Insets: Lattice fringes with distance of 2.05 Å corresponding to the Ru (101) crystal plane.]

Representative TEM images obtained from the reduced and spent Ru/SiO_2 samples are presented in Supplementary Fig. 9. The inset HRTEM images revealed an ordered crystal structure with interplanar distance of 2.05 Å, which was constant with that of the hcp (101) plane of metallic Ru. The average particle size of Ru NPs in unpromoted Ru/SiO_2 sample after reduction was close to 8 nm and a distribution of Ru NPs with size of 8.4 nm was observed for the spent Ru/SiO_2 catalyst. Comparing with Na-Ru/SiO_2 (Fig. 2b), the sodium promoter is beneficial to improve the dispersion of Ru NPs.

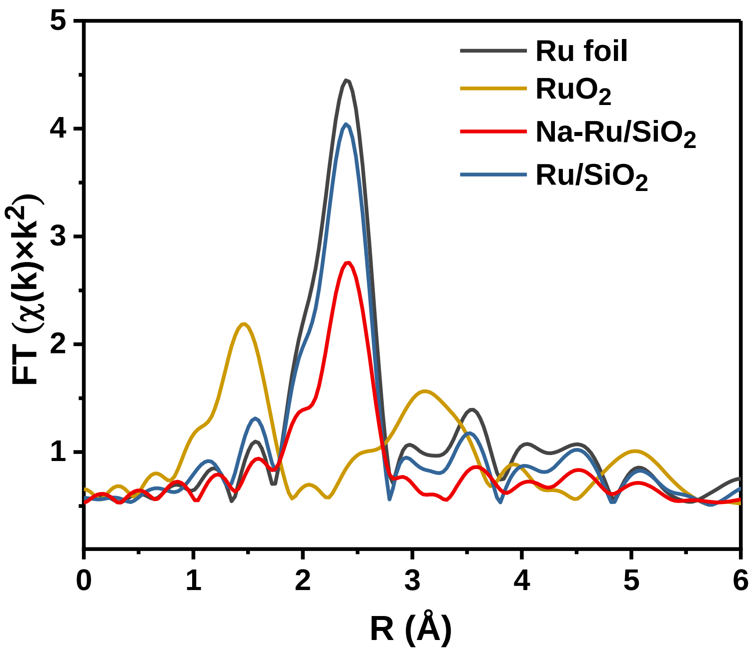

Supplementary Fig. 10 | EXAFS characterization. Fourier transforms of the k^3-weighted EXAFS of Ru K-edge for Ru foil, RuO_2, reduced Ru/SiO_2 and reduced $Na-Ru/SiO_2$ catalysts.

The Ru K-edge Fourier transformed EXAFS spectra of the reduced Ru/SiO_2 and $Na-Ru/SiO_2$ catalysts were substantially different from that of RuO_2 and very close to that of Ru foil. The strongest shell peak of the EXAFS spectra attributed to Ru-Ru pair ~2.4 Å appeared in both $Na-Ru/SiO_2$ and Ru/SiO_2 samples, indicating the existence of Ru metal phase for these two cases.

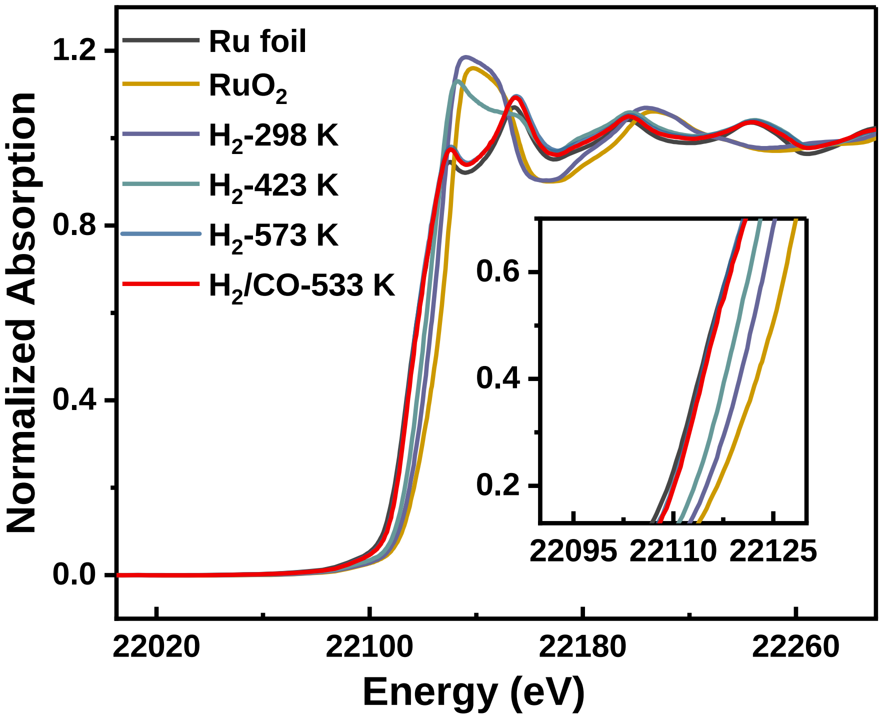

Supplementary Fig. 11 | In situ XANES characterization. Ru K-edge X-ray absorption near-edge structure (XANES) spectra of Na-Ru/SiO_2 sample during stepwise reduction (H_2-298 K → H_2-423 K→ H_2-573 K) and FTO reaction process (H_2/CO-533 K).

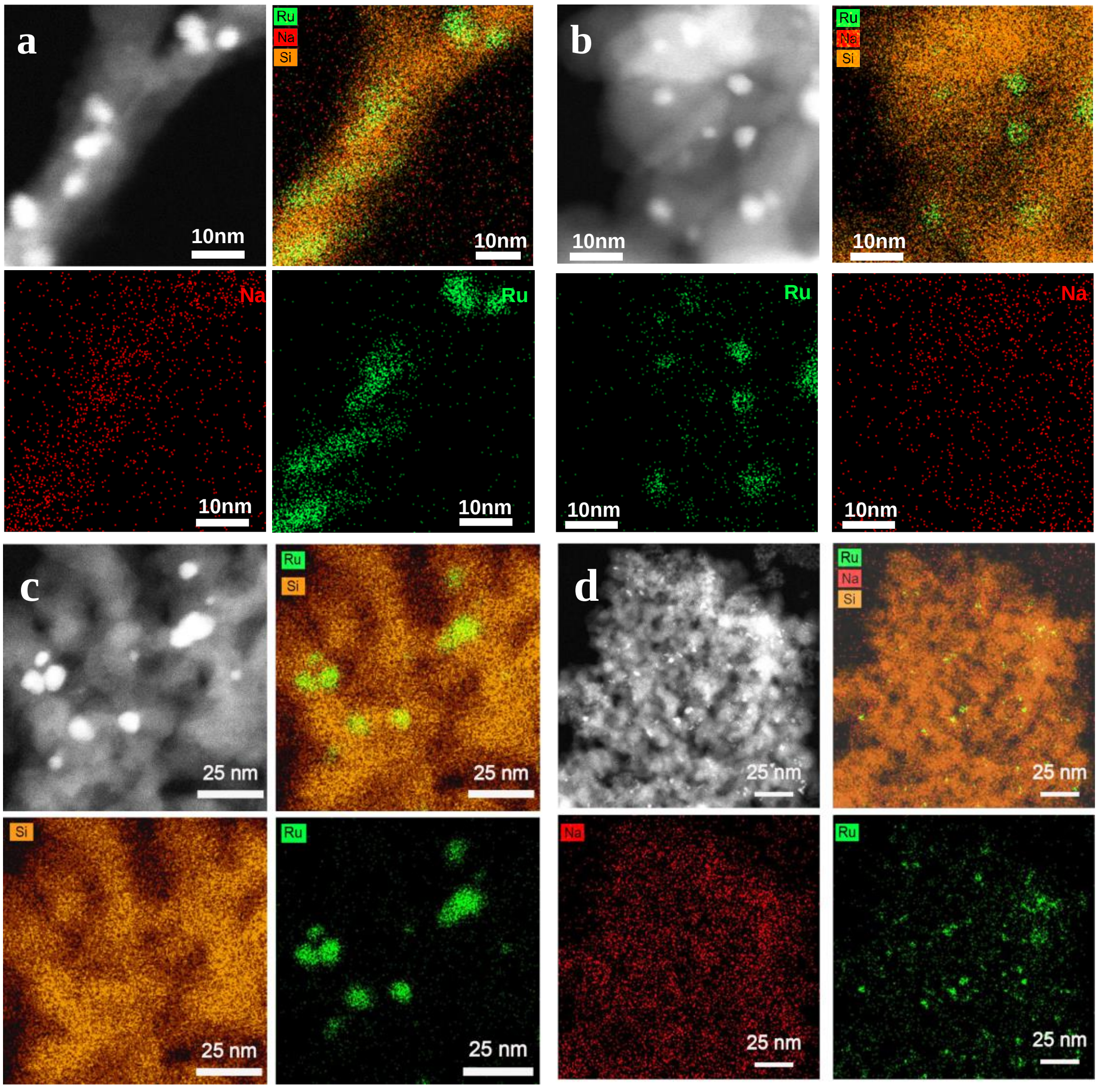

Supplementary Fig. 12 | HAADF-STEM characterization and EDX elemental mapping. (**a**) reduced Na-Ru/SiO_2, (**b**) spent Na-Ru/SiO_2, (**c**) spent Ru/SiO_2 and (**d**) spent 2Na-Ru/SiO_2 catalyst. Ru (green), Na (red), Si (orange).

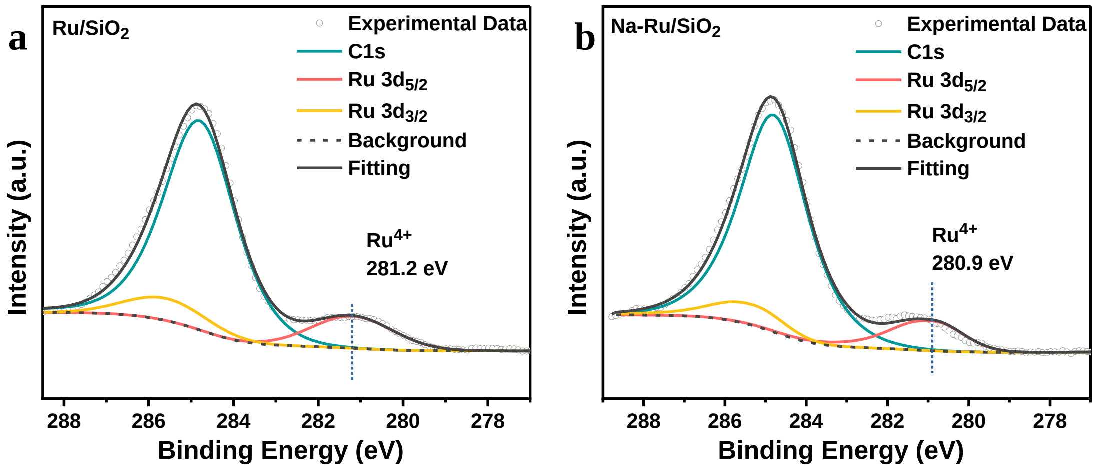

Supplementary Fig. 13 | XPS spectra. XPS profiles in the Ru 3*d* for Ru/SiO_2 (**a**) and Na-Ru/SiO_2 (**b**) after calcination in air atmosphere.

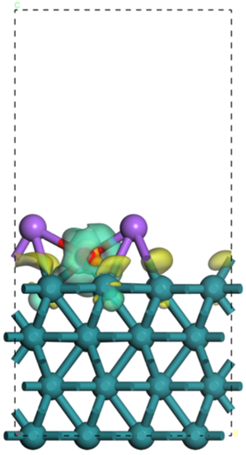

surface	Na_2O/Ru(0001)	Isolated Na_2O
Na_1(|e|)	6.17	6.26
Na_2(|e|)	6.19	6.26
O(|e|)	7.00	7.48
Total(|e|)	19.36	20.00
nElect Loss	0.64	

Supplementary Fig. 14 | Charge density difference calculation. (Left) The side views of Na_2O charge density difference for Na_2O/Ru (0001). Atoms outside the calculation unit cell are depicted as smaller spheres; purple is Na, red is O and loden is Ru. (Right) Calculated number of valence electrons from Bader charge analysis, where each Na atom has 4 core electrons ($1s^2 2s^2$) and each O atom has 2 core electrons ($1s^2$).

Charge density difference calculations showed electron transfer from the Na_2O cluster to the Ru (0001) surface. Bader charge analysis showed that for the isolated Na_2O cluster, the oxygen atom obtained electron from the coordinated Na atoms with the calculated Bader charge value of -1.48 |e|. When Na_2O was located on the Ru (0001) surface, electrons were predicted to be transferred from Na_2O to Ru substrate, and the Bader charge value of the oxygen atom was calculated to be -1.00 |e|. The above results suggested that the Na atoms lose electrons to O in Na_2O, and when Na_2O was anchored on the Ru (0001) surface, electrons accumulated at the O atom decreased due to electron transfer to Ru surface.

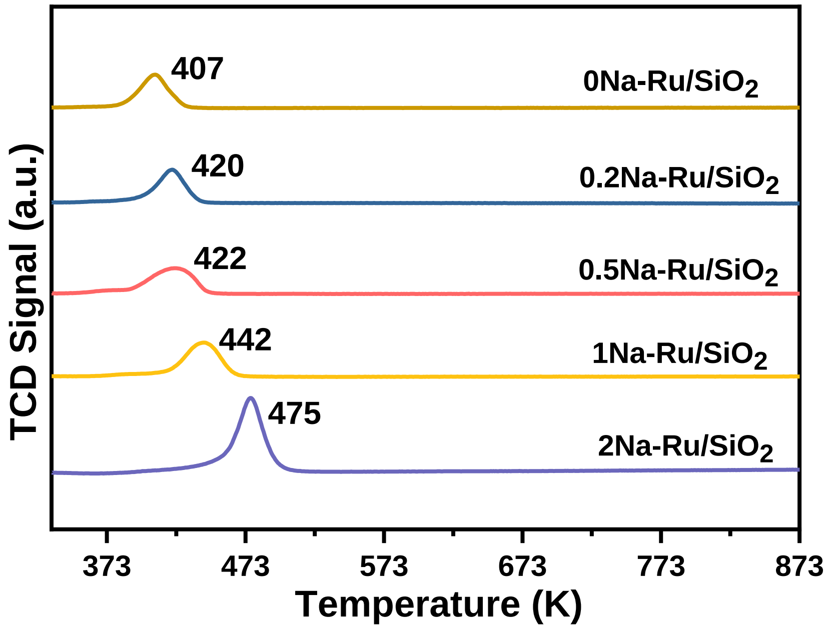

Supplementary Fig. 15 | H_2-TPR characterization. H_2-TPR profiles of the calcined Ru/SiO_2 catalysts with different Na/Ru molar ratio.

As shown in Supplementary Fig. 15, an obvious H_2 consumption peak was observed in H_2-temperature-programmed reduction (H_2-TPR) profiles, which is originated from the reduction of RuO_2 to metallic Ru. With the increase of doping amount of Na promoter, the reduction peak progressively shifted to higher temperature, suggesting that the sodium promoter greatly retarded the reduction of RuO_2.

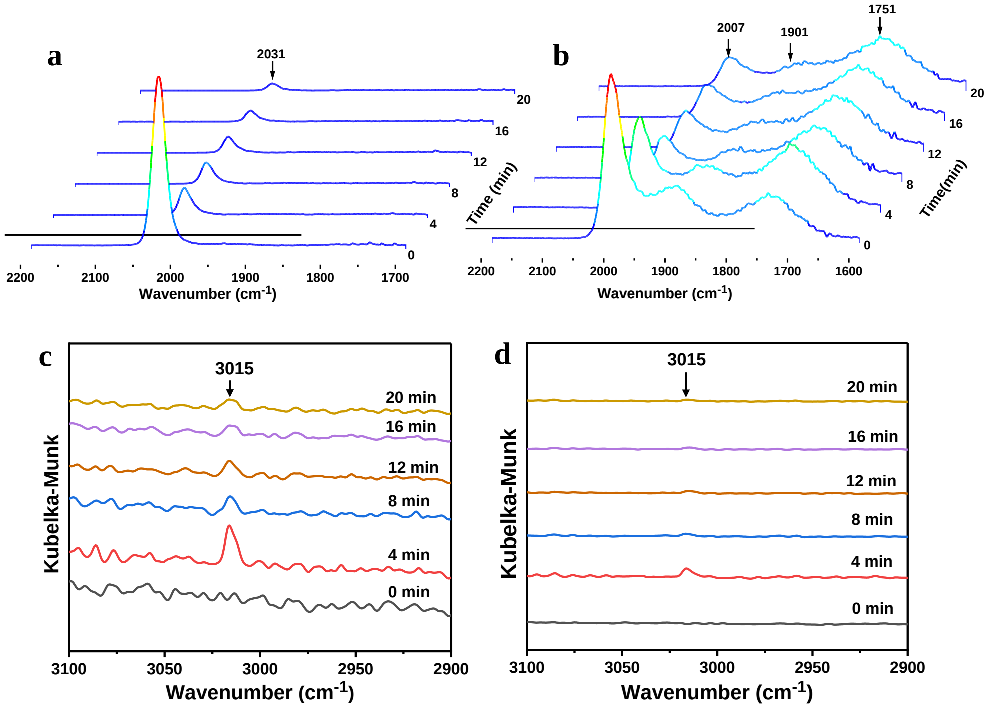

Supplementary Fig. 16 | In situ DRIFT spectra. Evolution of CO_{ad} species during H_2 flow at 533 K as determined using in situ DRIFT spectra over Ru/SiO_2 (**a**, **c**) and Na-Ru/SiO_2 (**b**, **d**) catalysts.

As shown in Supplementary Fig. 16, after the saturated-absorption of CO, the flow was switched to 25%H_2/Ar (10 mL min^{-1} H_2, 30 mL/min Ar) at 533 K. The evolution of CO-DRIFTS spectra at initial 20 min over Ru/SiO_2 (Supplementary Fig. 16 a) and Na-Ru/SiO_2 (Supplementary Fig. 16 b) was recorded. For these two cases, the consumption of CO_{ad} on metallic Ru NPs was clearly observed. Simultaneously, the characteristic peak of CH_4 at 3015 cm^{-1} gradually appeared and then finally disappeared (Supplementary Fig. 16 c~d), which derived from the hydrogenation of surface carbon species obtained via CO dissociation. However, the peak intensity of remaining CO_{ad} species over Na-Ru/SiO_2 was stronger than that of Ru/SiO_2, suggesting that the CO_{ad} species were strongly bonded on Ru surface of Na-Ru/SiO_2. Inversely, a much stronger peak intensity of CH_4 for Ru/SiO_2 than that of Na-Ru/SiO_2 confirmed that the surface carbon species tended to be hydrogenated over Ru/SiO_2. These results indicated that the Na doping can strengthen the CO adsorption capacity while suppressing the hydrogenation ability of Ru-based catalyst.

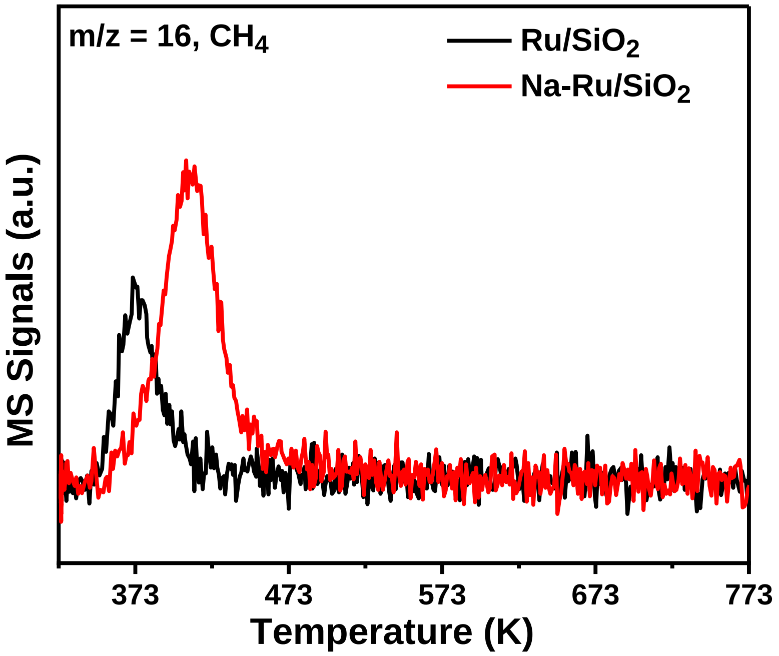

Supplementary Fig. 17 | CO-TPSR characterization. MS signals of methane during CO-TPSR test over Ru/SiO_2 and Na-Ru/SiO_2 catalysts.

As shown in Supplementary Fig. 17, the surface carbon species obtained via dissociation of CO_{ad} species can be hydrogenated to form CH_4 during temperature-programmed process under the flow of H_2. The preferential appearance of CH_4 signal suggested the hydrogenation ability of H_2 over Ru/SiO_2 was much stronger than that of Na-Ru/SiO_2. Simultaneously, the higher peak area and intensity of CH_4 signal for Na-Ru/SiO_2 confirmed more CO can be adsorbed and activated over the Na-doped samples[4], in line with the result obtained in Supplementary Fig. 16.

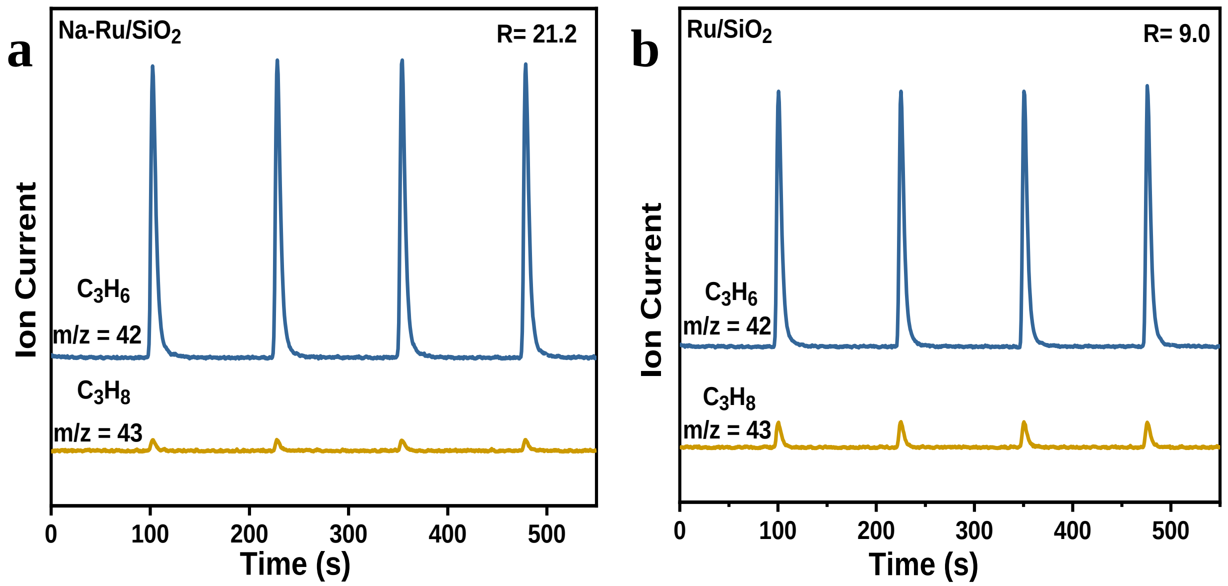

Supplementary Fig. 18 | Pulse experiment. Transient response curves obtained during pulses of C_3H_6 into a flow of diluted H_2 (10% H_2,90% Ar, 20 mL min^{-1}) at 533 K for Na-Ru/SiO_2 (**a**) and Ru/SiO_2 (**b**) catalysts. Before the pulse of C_3H_6, the samples were reduced by hydrogen at 723 K and then reacted in syngas at 533 K for 1 h. R denotes the integrated peak area ratio of C_3H_6/C_3H_8 detected by mass spectrometer.

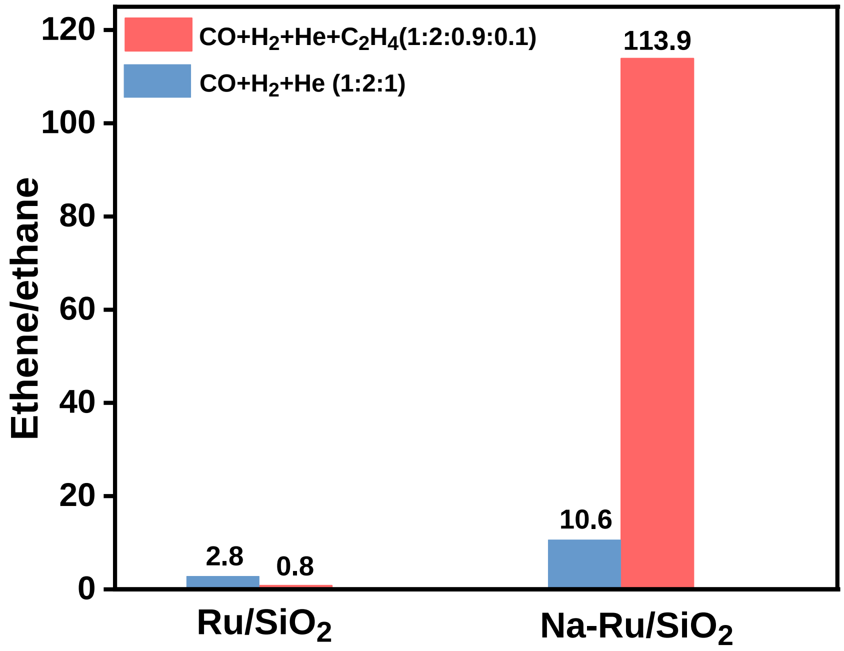

Supplementary Fig. 19 | Ethene co-feeding experiments. Comparison of ethene/ethane ratio before and after the addition of ethene to the feed gas over Ru/SiO_2 and Na-Ru/SiO_2 catalysts at 533 K, 1.0 MPa, H_2/CO ratio of 2.

As shown in Supplementary Fig. 19, the added ethene was totally hydrogenated to ethane over a working Ru/SiO_2 catalyst, resulting in a very low ratio of ethene/ethane (0.8). As for Na-Ru/SiO_2, however, the added ethene was almost remained, leading to a high ethene/ethane ratio up to 113.9. It was demonstrated that ethene readily desorbed on the Na-Ru/SiO_2, and the hydrogenation of ethene to ethane was also significantly hindered. Obviously, the reactivity of chemisorbed H_2 was greatly reduced with the addition of Na promoter into the Ru/SiO_2 catalyst.

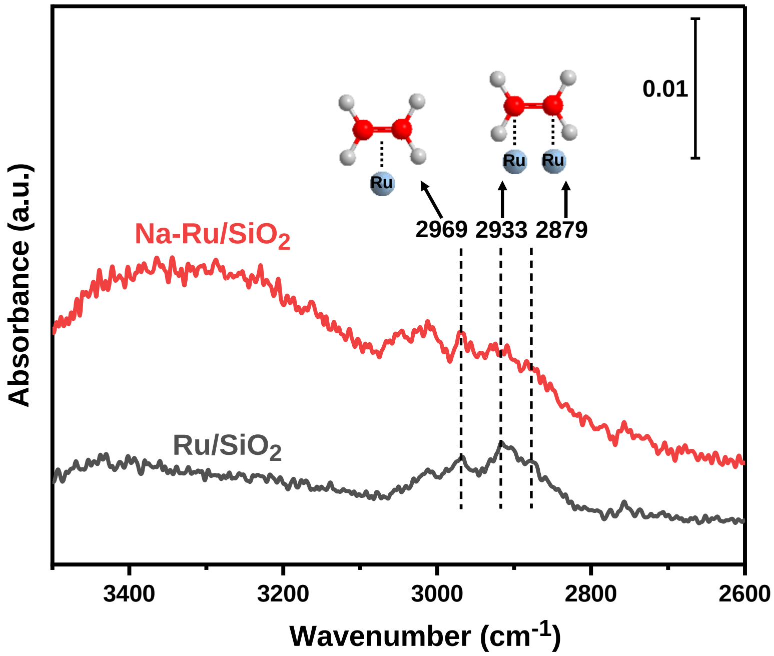

Supplementary Fig. 20 | IR spectra of C_2H_4 adsorption. Infrared study of C_2H_4 adsorbed on Ru/SiO_2 and $Na\text{-}Ru/SiO_2$ catalysts using DRIFTS at 533 K.

Supplementary Fig. 20 presents the DRIFT spectra of C_2H_4 adsorption. The peak at 2969 cm^{-1} was assigned to the π-bounded ethylene on Ru sites, and the peaks at 2933 and 2879 cm^{-1} were attributed to di-σ-bounded ethylene on Ru sites[5]. Obviously, the peaks intensity for di-σ-bounded ethylene over Ru/SiO_2 was stronger than that over $Na\text{-}Ru/SiO_2$, indicating that the introduction of Na promoter inhibited the adsorption of C_2H_4.

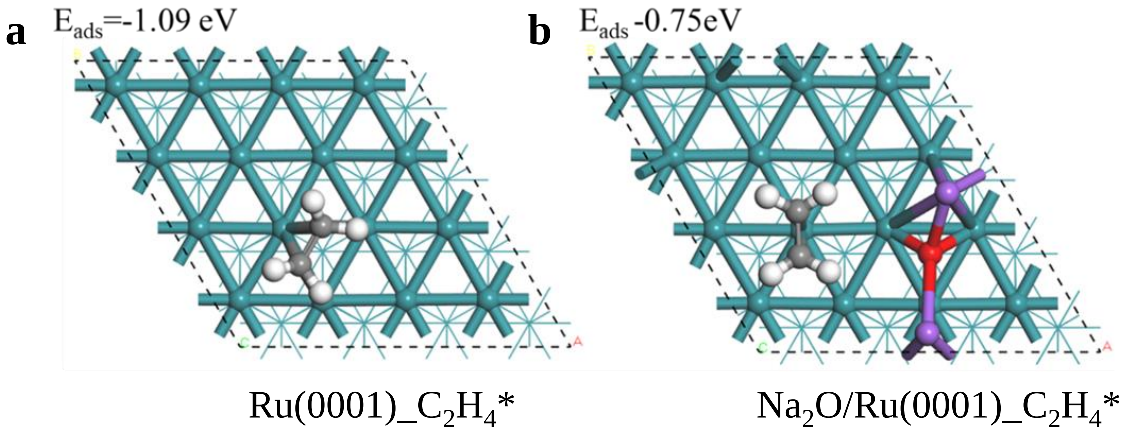

Supplementary Fig. 21 | Optimized geometries of adsorbed ethylene. (**a**) Ru (0001) surface, and (**b**) Na_2O/Ru (0001) surface.

The adsorption energies of ethylene chemisorption on the top Ru were calculated to be -1.09 eV and -0.75 eV before and after introducing Na_2O, respectively.

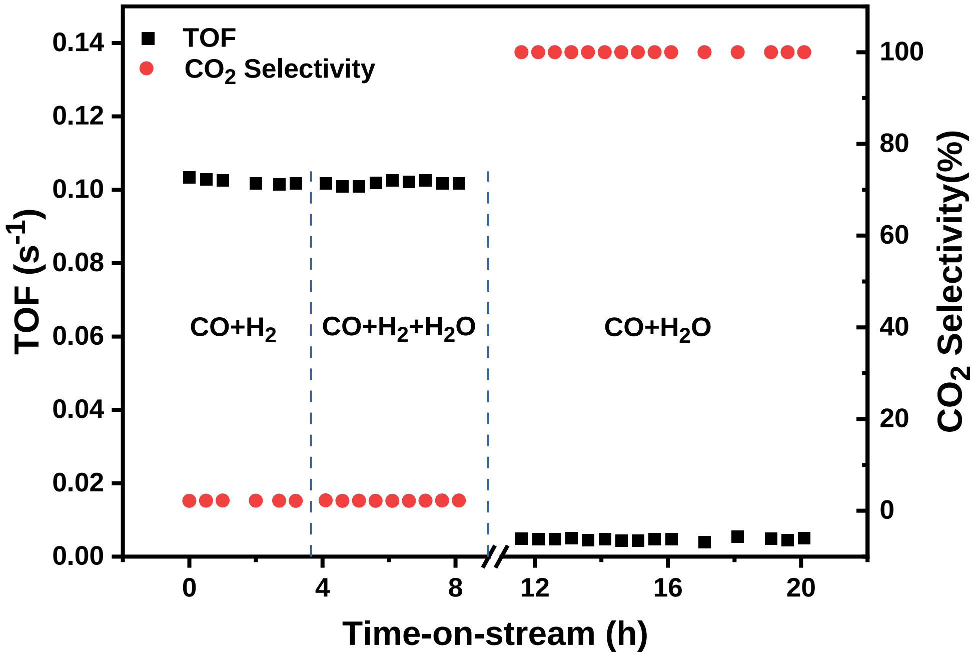

Supplementary Fig. 22 | Water-gas-shift probe reaction. The evolution of TOF and CO_2 selectivity over Na-Ru/SiO_2 catalyst before and after the introduction of H_2O into the reaction system at 533 K and 1 MPa.

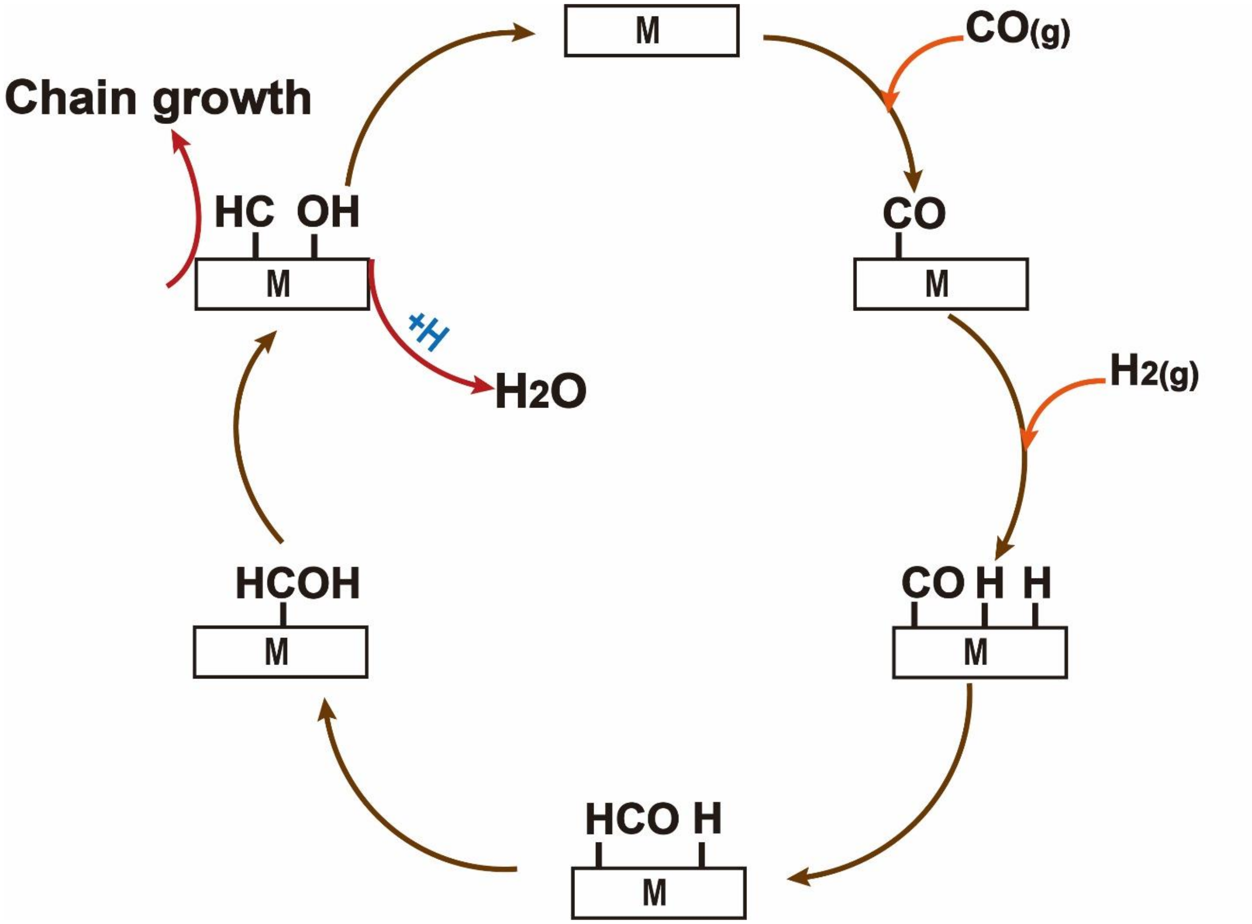

Supplementary Fig. 23 | CO activation mechanism. Elementary steps for H*-assisted CO* dissociation route on Ru surface[1].

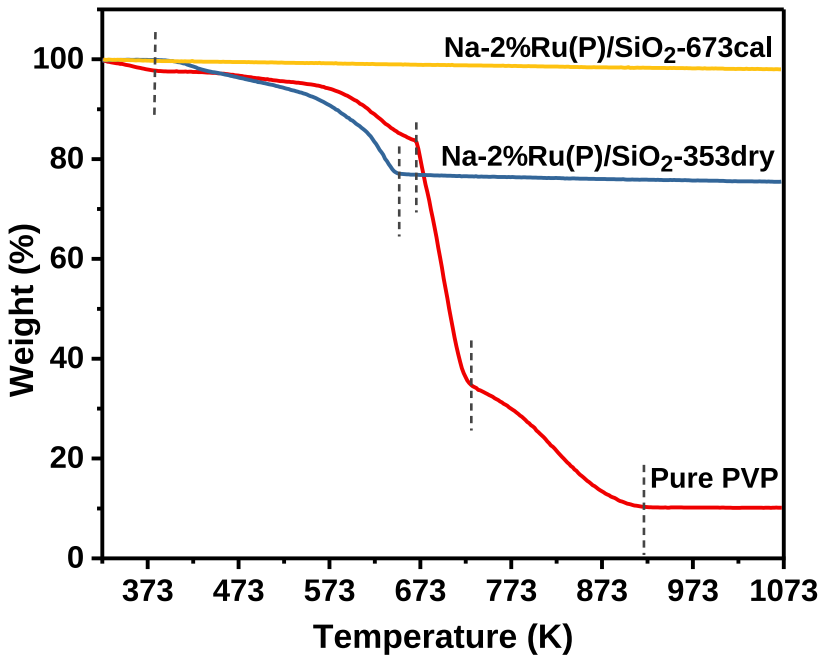

Supplementary Fig. 24 | Thermogravimetric (TG) curves. TG analysis of pure PVP, Na-2%Ru(P)/SiO_2 sample dried at 353 K (Na-2%Ru(P)/SiO_2-353dry) and Na-2%Ru(P)/SiO_2 sample calcined at 673 K (Na-2%Ru(P)/SiO_2-673cal).

Na-2%Ru(P)/SiO_2-353dry showed an obvious weight loss due to the consumption of PVP with the increase of temperature. However, for Na-2%Ru(P)/SiO_2-673cal, no weight loss can be observed, suggesting that PVP was completely removed during the calcination process.

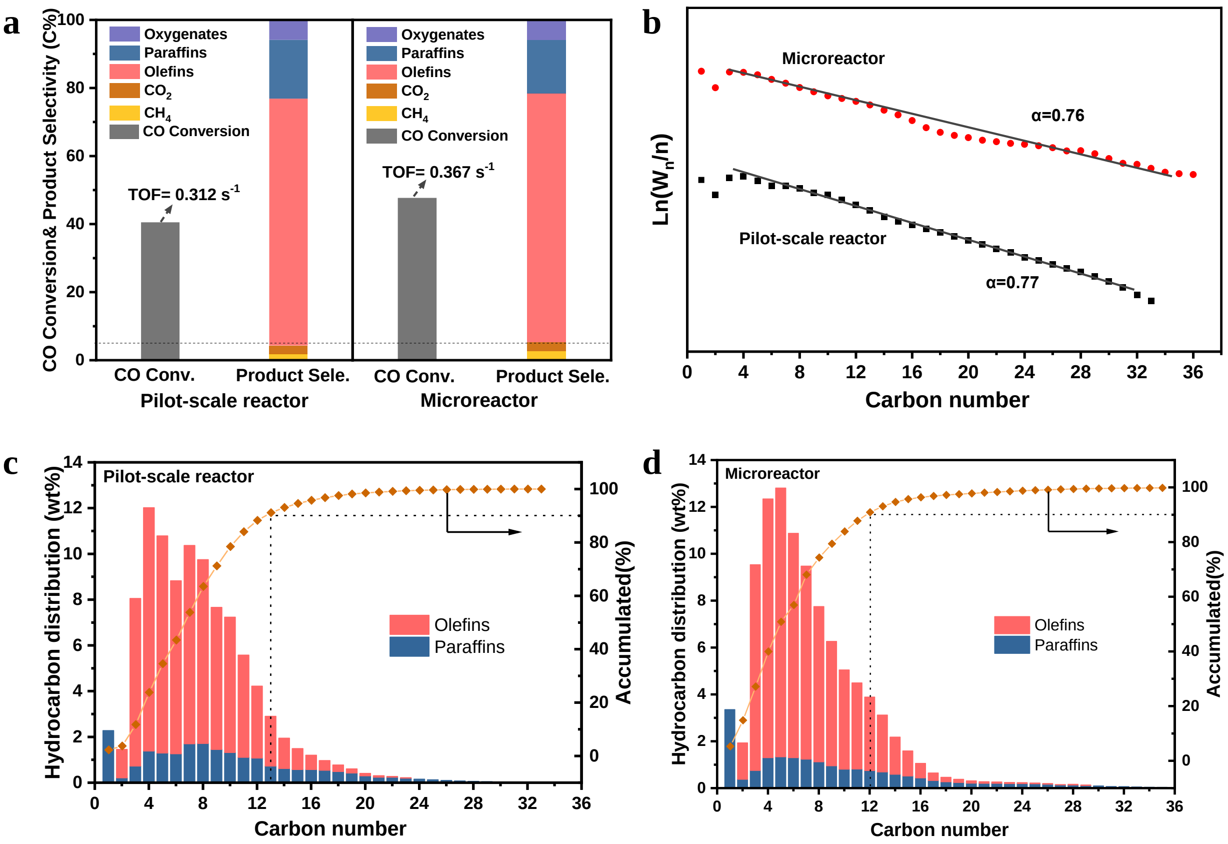

Supplementary Fig. 25 | Comparison of detailed catalytic results evaluated in a pilot-scale reactor and microreactor. (**a**) CO conversion and product selectivity. (**b**) ASF distribution and chain-growth probability (α) and (**c**, **d**) hydrocarbons distribution of the Na-2%Ru(P)/SiO_2 catalyst in the pilot-scale reactor (**c**) and microreactor (**d**). Reaction conditions: 538 K, 1.0 MPa, 3000 mL $g_{cat.}^{-1}$ h^{-1} and H_2/CO ratio of 2.

The Na-2%Ru(P)/SiO_2 catalyst was evaluated in a pilot-scale reactor (12 – 20 mesh) and microreactor (40 – 60 mesh), respectively, under reaction conditions of 538 K, 1.0 MPa, 3000 mL $g_{cat.}^{-1}$ h^{-1}, H_2/CO ratio of 2. As shown in Supplementary Fig. 25, the olefins selectivity in total products reached up to 72.5% while the sum selectivity of undesired CH_4 and CO_2 was suppressed within 5% at CO conversion of 40.5% and TOF of 0.312 s^{-1} in the pilot-scale reactor, which is very similar to that in microreactor. The CH_4 selectivity for both reactors were much lower than the value predicated by the classic ASF model. Moreover, a chain-growth probability at around 0.76 was obtained in both pilot-scale reactor and microreactor, demonstrating the as-obtained catalyst is very suitable to produce long-chain olefins. In addition, a similar hydrocarbon distribution was also obtained, confirming that the pellet Na-2%Ru(P)/SiO_2 catalyst shows a

promising industrial application with high olefins yield and low fraction of undesired C1 by-products.

Supplementary Tables

Supplementary Table 1. Comparison of catalytic performance with previous works.

Entry	Catalyst category	Catalyst	T (K)	P (MPa)	H_2/CO ratio	WHSV (mL $g_{cat.}^{-1}$ h^{-1})	CO Conv. (%)	Reaction Rate (mol_{CO} $g_{cat.}^{-1}$ h^{-1})	Product Selectivity (%)					Olefins yield (%)	Ref.
									CO_2	CH_4	C1(CO_2+CH_4)	Olefins	Others		
1	Oxide-Zeolite	$ZnCrO_x$/MSAPO	673	2.5	2.5	5143	17.0	0.0112	41.0	1.2	42.2	47.2[c]	10.6	8.0[c]	*(6)*
2		$ZnZrO_x$/SAPO	673	1	2	3600	9.5	0.0051	45.0	6.0	51.0	34.7[c]	14.3	3.3[c]	*(7)*
3		$ZnCrO_x$/MOR	673	2.5	2.5	1857	12.0	0.0028	45.0	2.8	47.8	44.0[c]	8.2	5.3[c]	*(8)*
4		$ZnAl_2O_4$|SAPO-34	663	4	1	12000	6.9	0.0185	33.1	3.7	36.8	51.5[c]	11.7	3.6[c]	*(9)*
5		MnO_x/SAPO	673	2.5	2.5	4800	8.5	0.0052	41.0	2.0	43.0	46.7[c]	10.3	4.0[c]	*(10)*
6	Fe-based	Fe-Zn-0.81Na	613	2	2.7[a]	60000	77.2	0.5589	23.0	9.7	32.7	60.2	7.1	46.5	*(11)*
7		FeMn@Si-c	593	3	2	4000	56.1	0.0334	13.0	10.0	23.0	65.3	11.7	36.6	*(12)*
8		Fe/α-Al_2O_3	613	2	1	1500[b]	80.0	-	40.0	6.6	46.6	31.8[c]	21.6	25.4[c]	*(13)*
9		Fe-K/NCNTs	573	0.1	1	4200	16.5	0.0155	23.6	17.3	40.9	41.7[c]	17.4	6.9[c]	*(14)*
10		Fe/hNCNC	623	0.1	1	12000	3.5	0.0094	39.4	25.0	64.4	32.8[c]	2.8	1.1[c]	*(15)*
11		Mn/Fe_3O_4	593	1	1	4480	41.5	0.0415	37.8	9.7	47.5	37.4[c]	15.1	15.5[c]	*(16)*
12		Fe10In/Al_2O_3	673	0.5	2	7800	11.0	0.0128	16.0	~22.0	~38.0	45.0[c]	~17.0	5.0[c]	*(17)*
13		Fe_3O_4@MnO_2	553	2	1	3000	67.9	0.0455	47.1	3.6	50.7	41.9	7.4	28.5	*(18)*
14	Co-based	CoMn	523	0.1	2	2000	31.8	0.0095	47.3	2.6	49.9	32.0[c]	18.1	10.2[c]	*(19)*
15		Co_1Mn_3-Na_2S	513	0.1	2	-	0.8	-	< 3.0	17.0	< 20.0	54.0[c]	26.0	0.4[c]	*(20)*
16		0.5Na/CoMnAl@6.6Si	533	1	0.5	4000	13.5	0.0161	16.7	4.3	21.0	61.1	17.9	8.2	*(21)*
17		1.0Pr-CoRu/AOmM	473	2	2	-	20±3	-	0.9	8.4	9.3	19.9[d]	-	-	*(22)*
18	Ru-based	Na-5%Ru/SiO_2	533	1	2	3000	45.8	0.0204	2.7	2.2	4.9	80.1	15.0	36.7	This work
19		Na-5%Ru/SiO_2	533	1	2	1500	67.9	0.0152	2.7	4.0	6.7	76.6	16.7	51.9	
20		Na-5%Ru(P)/SiO_2	533	1	2	3000	65.3	0.0292	2.7	1.9	4.6	73.7	21.7	48.1	

[a] 8 C% of CO_2 is included in the syngas feedstock (CO:H_2:CO_2:Ar=24:64:8:4).

[b] GHSV of 1500 h^{-1} is used.

[c] The values denote the selectivity and yield of lower olefins ($C_{2-4}^{=}$).

[d] C_5-C_{11} olefins.

Supplementary Table 2. Effect of H_2/CO ratio on catalytic performance of Na-Ru/SiO_2.[a]

H_2/CO ratio	CO Conv. (%)	Selectivity (C %)				Yield (%)
		Olefins	C_{2+} paraffins[b]	CO_2	CH_4	
0.5	6.8	77.0	16.6	4.4	2.0	5.2
1	15.2	76.4	17.0	4.2	1.6	11.6
2	45.8	80.1	15.0	2.7	2.2	36.7
4	61.0	72.6	19.7	2.6	5.1	44.3
5	72.4	69.7	22.5	2.1	5.7	50.5

[a] Reaction condition: 1 MPa, 533 K, 3000 $mL \cdot g_{cat.}^{-1} \cdot h^{-1}$.

[b] Paraffins with two or more carbon atoms.

Supplementary Table 3. Effect of space velocity on catalytic performance of Na-Ru/SiO_2.[a]

WHSV ($mL\ g_{cat.}^{-1}\ h^{-1}$)	CO Conv. (%)	Selectivity (C %)				Yield (%)
		Olefins	C_{2+} paraffins[b]	CO_2	CH_4	
1500	67.9	76.6	16.7	2.7	4.0	51.9
3000	45.8	80.1	15.0	2.7	2.2	36.7
6000	13.1	78.3	14.3	3.0	4.4	10.2
9000	7.5	78.6	13.4	3.3	4.7	5.9

[a] Reaction condition: 1 MPa, 533 K, H_2/CO=2.

[b] Paraffins with two or more carbon atoms.

Supplementary Table 4. Effect of reaction pressure and space velocity on catalytic performance of Na-Ru /SiO_2 catalyst. [a]

Pressure (MPa)	WHSV (mL $g_{cat.}^{-1}$ h^{-1})	CO Conv. (%)	Selectivity (C %)					Yield (%)
			Oxy.[b]	Olefins	C_{2+} paraffins[c]	CO_2	CH_4	
0.5	3000	12.7	0.1	81.0	9.1	5.0	4.9	10.3
1	3000	45.8	0.5	79.7	14.9	2.7	2.2	36.5
2	3000	68.5	1.9	55.5	38.1	1.3	3.2	39.3
2	6000	35.5	1.6	62.4	32.4	0.9	2.7	22.9
3	6000	49.5	13.9	52.2	30.3	0.9	2.7	30.0
3	9000	31.2	11.5	53.5	32.0	0.7	2.3	18.9

[a] Reaction conditions: 533 K, and a H_2/CO ratio of 2.

[b] Oxygenates including alcohols and aldehydes.

[c] Paraffins with two or more carbon atoms.

Supplementary Table 5. Effect of reaction temperature on catalytic performance of Na-Ru/SiO_2.[a]

Temperature (K)	CO Conv. (%)	Selectivity (C %)				Yield (%)
		Olefins	C_{2+} paraffins[b]	CO_2	CH_4	
493	13.8	69.3	27.6	0.4	2.7	9.6
513	26.2	69.9	28.9	0.8	3.0	18.3
533	45.8	80.1	15.0	2.7	2.2	36.7
553	60.9	73.9	18.2	5.0	2.9	45.0

[a] Reaction condition: 1 MPa, H_2/CO=2, 3000 mL·$g_{cat.}^{-1}$·h^{-1}.

[b] Paraffins with two or more carbon atoms.

Supplementary Table 6. Catalytic performance of the Ru/SiO_2 catalysts with different alkali promoters. [a]

Catalyst	CO Conv. (%)	Selectivity (C %)				Olefin distribution (C%)		Yield (%)
		Olefins	C_{2+} paraffins[b]	CO_2	CH_4	$C_{2-4}^{=}$	$C_{5+}^{=}$	
0Na-Ru/SiO_2	73.3	16.9	76.5	0.3	6.4	31.7	68.3	12.4
0.5Li-Ru/SiO_2	51.3	75.6	19.2	1.5	3.8	27.3	72.7	38.8
0.5Na-Ru/SiO_2	45.8	80.1	15.0	2.7	2.2	25.5	74.5	36.7
0.5K-Ru/SiO_2	42.2	74.4	22.0	1.8	1.8	19.6	80.4	31.4
0.5Rb-Ru/SiO_2	40.1	72.7	22.9	2.2	2.2	17.4	82.6	29.2
0.5Cs-Ru/SiO_2	32.0	60.5	34.0	2.6	2.9	17.0	83.0	19.4

[a] Reaction conditions: 533 K, 1.0 MPa, 3000 mL $g_{cat.}^{-1}$ h^{-1}, and H_2/CO ratio of 2.

[b] Paraffins with two or more carbon atoms.

Supplementary Table 7. CO chemisorption and ICP results for different Ru-based catalysts after reduction.

Sample	Ru loading[a] (wt.%)	Na loading[a] (wt.%)	Na/Ru molar ratio	d_{XRD}[b] (nm)	d_{TEM}[c] (nm)	D_{TEM}[d] (%)	CO uptake (μmol•g^{-1})	Metallic Surface Area (m^2•g_{Ru}^{-1})	D_{CO}[e] (%)
Ru/SiO_2	4.57	0.06	0.06	7.8	7.9	14.2	24.0	23.8	5.3
$Na-Ru/SiO_2$	4.18	0.57	0.60	5.3	4.7	23.8	45.5	49.3	11.0
$Na-5\%Ru(P)/SiO_2$	4.11	0.55	0.58	6.2	4.4	25.5	51.4	56.7	12.7
$Na-2Ru(P)/SiO_2$	1.79	0.21	0.52	6.2	5.4	20.7	16.1	40.8	9.1

[a] Ru loading and Na loading measured by ICP.

[b] Ru^0 crystallites size calculated by Scherer Formula from XRD.

[c] Ru^0 mean particle size counted by TEM profiles.

[d] $D_{TEM}=1.12/d_{TEM}$.

[e] Dispersion of Ru^0 nanoparticles calculated by CO chemisorption experiment.

Supplementary Table 8. Structural parameters of the reduced catalysts, Ru foil and RuO_2 standard sample from the EXAFS fitting (S_0^2=0.75). [a]

Sample	Shell	[b]CN	[c]R(Å)	[d]$\sigma^2\times10^2$(Å^2)	[e]ΔE_0(eV)	R factor
Ru foil	Ru-Ru	12	2.67±0.01	0.35±0.03	1.36±1.16	0.013
RuO_2	Ru-O	6	1.97±0.01	0.24±0.08	3.79±1.67	0.010
Ru/SiO_2	Ru-Ru	10.5	2.67±0.01	0.37±0.07	1.13±1.07	0.011
Na-Ru/SiO_2	Ru-Ru	9.2	2.66±0.01	0.59±0.06	2.00±0.61	0.007

[a] S_0^2 was fixed at 0.75 during EXAFS fitting, based on the known structure of the Ru foil.

[b] CN is the coordination number for the absorber-backscatter pair.

[c] R is the average interatomic distance.

[d] σ^2 is the Debye-Waller factor.

[e] ΔE_0 is the inner potential correction.

Supplementary Table 9. Catalytic results of different Na-promoted Ru-based catalysts with and without the addition of PVP for syngas conversion. [a]

Catalyst	CO Conv. (%)	Selectivity (C %)				Reaction Rate ($mol_{CO}\ g_{Ru}^{-1}\ h^{-1}$)
		Olefins	C_{2+} paraffins[b]	CO_2	CH_4	
Na-5%Ru/SiO_2	45.8	80.1	15.0	2.7	2.2	0.472
Na-5%Ru(P)/SiO_2	65.3	73.7	21.7	2.7	1.9	0.702
Na-2%Ru/SiO_2	16.3	80.9	13.9	2.8	2.5	0.503
Na-2%Ru(P)/SiO_2	27.6	77.7	18.2	2.1	2.0	0.688
Na-1%Ru/SiO_2	5.5	76.7	16.7	4.4	2.2	0.234
Na-1%Ru(P)/SiO_2	7.0	75.9	18.2	3.3	2.6	0.302

[a] Reaction conditions: 533 K, 1.0 MPa, 3000 mL $g_{cat.}^{-1}\ h^{-1}$, and a H_2/CO ratio of 2.

[b] Paraffins with two or more carbon atoms.

Supplementary Table 10. Comparison of the catalytic performance of various supported Ru-based catalysts during FTS reaction process.

Entry	Catalyst	T (K)	P (MPa)	Reaction Rate ($mol_{CO}\cdot g_{Ru}^{-1}\cdot h^{-1}$)	TOF (s^{-1})	C_{5+} Selectivity (C%)	Olefins Selectivity (C%)	Ref.
1	Na-Ru/SiO_2	533	1	0.472	0.126	77.0	80.1	This work
2	Na-5%Ru(P)/SiO_2	533	1	0.702	0.156	81.1	70.5	
3	Na-2%Ru(P)/SiO_2 -Microreactor	538	1	1.190	0.367	75.1	73.2	
4	Na-2%Ru(P)/SiO_2 -Pilot-scale reactor	538	1	1.010	0.312	83.8	72.5	
5	Ru/TiO_2-450R	433	2	0.473	0.039	80.0	-	(23)
6	Ru/TiO_2(R)	523	0.1	0.547	-	46.8	-	(24)
7	Ru/Al_2O_3-PHR	423	3	0.129	0.006	-	-	(25)
8	Ru/Al_2O_3-10Cl	523	4.04	0.333	0.030	75.0	-	(26)
9	Ru/C	503	2	0.077	-	63.2	-	(27)
10	Ru@Si/Al-50	543	2	0.404	-	68.0	-	(28)
11	Ru/meso-ZSM-5	533	2	0.507	0.067	69.8	-	(29)
12	Ru/meso-beta	533	2	0.531	0.071	54.0	-	(30)
13	Ru/HB-S	533	1	-	0.129	72.0	-	(31)
14	Ru/CNT	533	2	0.583	0.193	82.9	-	(32)
15	Ru@MHCS	523	1	0.342	0.023	56.3	-	(33)
16	Ru/TiO_2-500-H	493	2	-	0.047	72.6	-	(34)
17	Ru-in/TNT	513	2	0.216	-	64.7	-	(35)
18	10Ru/G100	513	1.5	0.689	-	65.0	-	(36)
19	3%Ru/CNT	503	1	0.320	-	76.5	-	(37)

Supplementary References:

1 Loveless, B. T., Buda, C., Neurock, M. & Iglesia, E. CO chemisorption and dissociation at high coverages during CO hydrogenation on Ru catalysts. *J. Am. Chem. Soc.* **135**, 6107-6121, (2013).

2 Filot, I. A. W., van Santen, R. A. & Hensen, E. J. M. Quantum chemistry of the Fischer–Tropsch reaction catalysed by a stepped ruthenium surface. *Catal. Sci. Technol.* **4**, 3129-3140, (2014).

3 Filot, I. A., van Santen, R. A. & Hensen, E. J. The optimally performing Fischer-Tropsch catalyst. *Angew. Chem. Int. Ed.* **53**, 12746-12750, (2014).

4 Sakakini, B. H. & Verbrugge, A. S. Temperature-programmed surface reaction as a means of characterizing supported-metal catalysts and probing their surface reactivity. *J. Chem. Soc., Faraday Trans.* **93**, 1637-1640, (1997).

5 An, J. *et al.* Acid-promoter-free ethylene methoxycarbonylation over Ru-clusters/ceria: the catalysis of interfacial Lewis acid–base pair. *J. Am. Chem. Soc.* **140**, 4172-4181, (2018).

6 Jiao, F. *et al.* Selective conversion of syngas to light olefins. *Science* **351**, 1065-1068, (2016).

7 Cheng, K. *et al.* Direct and highly selective conversion of synthesis gas into lower olefins: design of a bifunctional catalyst combining methanol synthesis and carbon–carbon coupling. *Angew. Chem. Int. Ed.* **55**, 4725-4728, (2016).

8 Jiao, F. *et al.* Shape-selective zeolites promote ethylene formation from syngas via a ketene intermediate. *Angew. Chem. Int. Ed.* **57**, 4692-4696, (2018).

9 Ni, Y. *et al.* Realizing and recognizing syngas-to-olefins reaction via a dual-bed catalyst. *ACS Catal.* **9**, 1026-1032, (2018).

10 Zhu, Y. *et al.* Role of manganese oxide in syngas conversion to light olefins. *ACS Catal.* **7**, 2800-2804, (2017).

11 Zhai, P. *et al.* Highly tunable selectivity for syngas-derived alkenes over zinc and sodium-modulated Fe_5C_2 catalyst. *Angew. Chem. Int. Ed.* **55**, 9902-9907, (2016).

12 Xu, Y. *et al.* A hydrophobic FeMn@Si catalyst increases olefins from syngas by suppressing C1 by-products. *Science* **371**, 610-613, (2021).

13 Galvis, H. M. T. *et al.* Supported iron nanoparticles as catalysts for sustainable production of lower olefins. *Science* **335**, 835-838, (2012).

14 Lu, J. *et al.* Promotion effects of nitrogen doping into carbon nanotubes on supported iron Fischer–Tropsch catalysts for lower olefins. *ACS Catal.* **4**, 613-621, (2014).

15 Zhuo, O. *et al.* Stabilizing the active phase of iron-based Fischer-Tropsch catalysts for lower olefins: mechanism and strategy. *Chem. Sci.* **10**, 6083-6090, (2019).

16 Liu, Y., Chen, J.-F., Bao, J. & Zhang, Y. Manganese-modified Fe_3O_4 microsphere catalyst with effective active phase of forming light olefins from syngas. *ACS Catal.* **5**, 3905-3909, (2015).

17 He, Y., Shi, H., Johnson, O., Joseph, B. & Kuhn, J. N. Selective and stable In-promoted Fe catalyst for syngas conversion to light olefins. *ACS Catal.* **11**, 15177-15186, (2021).

18 Wang, J. *et al.* Directly converting syngas to linear alpha-olefins over core-shell Fe_3O_4@MnO_2 catalysts. *ACS Appl. Mater. Interfaces* **10**, 43578-43587, (2018).

19 Zhong, L. *et al.* Cobalt carbide nanoprisms for direct production of lower olefins from syngas. *Nature* **538**, 84-87, (2016).

20 Xie, J. *et al.* Promoted cobalt metal catalysts suitable for the production of lower olefins from natural gas. *Nat. Commun.* **10**, 167, (2019).

21 Lin, T. *et al.* Designing silica-coated CoMn-based catalyst for Fischer-Tropsch synthesis to olefins with low CO_2 emission. *Appl. Catal. B:Environ.* **299**, 120683, (2021).

22 Jeske, K. *et al.* Design of cobalt Fischer-Tropsch catalysts for the combined production of liquid fuels and olefin chemicals from hydrogen-rich syngas. *ACS Catal.* **11**, 4784-4798, (2021).

23 Zhang, Y. *et al.* Tuning reactivity of Fischer-Tropsch synthesis by regulating TiO_x overlayer over Ru/TiO_2 nanocatalysts. *Nat. Commun.* **11**, 3185, (2020).

24 Kikuchi, E., Matsumoto, M., Takahashi, T., Machino, A. & Morita, Y. Fischer-Tropsch synthesis over titania-supported ruthenium catalysts. *Appl. Catal.* **10**, 251-260, (1984).

25 Lian, C., Yu, Y., Zhang, K., Gao, A. & Wang, Y. Highly efficient Fischer–Tropsch synthesis over an alumina-supported ruthenium catalyst. *Catal. Sci. Technol.* **8**, 1528-1534, (2018).

26 González-Carballo, J. M. *et al.* In-situ study of the promotional effect of chlorine on the Fischer–Tropsch synthesis with Ru/Al_2O_3. *J. Catal.* **332**, 177-186, (2015).

27 Koh, T., Koo, H. M., Yu, T., Lim, B. & Bae, J. W. Roles of ruthenium–support interactions of size-controlled ruthenium nanoparticles for the product distribution of Fischer–Tropsch synthesis. *ACS Catal.* **4**, 1054-1060, (2014).

28 Hwang, J. *et al.* Direct confinement of Ru nanoparticles inside nanochannels of large pore mesoporous aluminosilicate for Fischer–Tropsch synthesis. *J. Mater. Chem. A* **3**, 23725-23731, (2015).

29 Kang, J. *et al.* Mesoporous zeolite-supported ruthenium nanoparticles as highly selective Fischer-Tropsch catalysts for the production of C_5-C_{11} isoparaffins. *Angew. Chem. Int. Ed.* **50**, 5200-5203, (2011).

30 Cheng, K. *et al.* Mesoporous beta zeolite-supported ruthenium nanoparticles for selective conversion of synthesis gas to C_5–C_{11} isoparaffins. *ACS Catal.* **2**, 441-449, (2012).

31 Sun, J. *et al.* Highly-dispersed metallic Ru nanoparticles sputtered on H-Beta zeolite for directly converting syngas to middle isoparaffins. *ACS Catal.* **4**, 1-8, (2013).

32 Kang, J., Zhang, S., Zhang, Q. & Wang, Y. Ruthenium nanoparticles supported on carbon nanotubes as efficient catalysts for selective conversion of synthesis gas to diesel fuel. *Angew. Chem. Int. Ed.* **48**, 2565-2568, (2009).

33 Phaahlamohlaka, T. N., Kumi, D. O., Dlamini, M. W., Jewell, L. L. & Coville, N. J. Ruthenium nanoparticles encapsulated inside porous hollow carbon spheres: A novel catalyst for Fischer–Tropsch synthesis. *Catal. Today* **275**, 76-83, (2016).

34 Lyu, S. *et al.* Dopamine sacrificial coating strategy driving formation of highly active surface-exposed Ru sites on Ru/TiO_2 catalysts in Fischer–Tropsch synthesis. *Appl. Catal. B:Environ.* **278**, 119261, (2020).

35 Yang, X. *et al.* Effect of confinement of TiO_2 nanotubes over the Ru nanoparticles on Fischer-Tropsch synthesis. *Appl. Catal. A:Gen.* **526**, 45-52, (2016).

36 Eslava, J. L., Sun, X., Gascon, J., Kapteijn, F. & Rodr ǵuez-Ramos, I. Ruthenium particle size and cesium promotion effects in Fischer–Tropsch synthesis over high-surface-area graphite supported catalysts. *Catal. Sci. Technol.* **7**, 1235-1244, (2017).

37 Xiong, K., Li, J., Liew, K. & Zhan, X. Preparation and characterization of stable Ru nanoparticles embedded on the ordered mesoporous carbon material for applications in Fischer–Tropsch synthesis. *Appl. Catal. A:Gen.* **389**, 173-178, (2010).
